# Shift Schedule With Fewer Short Daily Rest Periods and Sickness Absence Among Health Care Workers: A Cluster Randomized Clinical Trial

**DOI:** 10.1001/jamanetworkopen.2025.31568

**Published:** 2025-09-15

**Authors:** Ingebjørg Louise Rockwell Djupedal, Anette Harris, Erling Svensen, Stein Atle Lie, Astrid Louise Hanssen Wang, Ståle Pallesen, Siri Waage, Morten Birkeland Nielsen, Erlend Sunde, Bjørn Bjorvatn, Øystein Holmelid, Øystein Vedaa

**Affiliations:** 1 Department of Psychosocial Science, University of Bergen, Bergen, Norway; 2 Department of Health Promotion, Norwegian Institute of Public Health, Bergen, Norway; 3 Department of Research, Division of Psychiatry, Haukeland University Hospital, Bergen, Norway; 4 Department of Human Resources, Haukeland University Hospital, Bergen, Norway; 5 Department of Clinical Dentistry, University of Bergen, Bergen, Norway; 6 Department of Economics, University of Bergen, Bergen, Norway; 7 Norwegian Competence Centre for Sleep Disorders, Haukeland University Hospital, Bergen, Norway; 8 Department of Global Public Health and Primary Care, University of Bergen, Bergen, Norway; 9 Department of Work Psychology and Physiology, National Institute of Occupational Health, Oslo, Norway

## Abstract

**Importance:**

Some shift work arrangements allow for less than 11 hours off between shifts. The consequences of short daily rest periods are currently not well understood.

**Objective:**

To determine the effect and cost-benefit of reducing the number of short daily rest periods on sickness-related absence among health care workers.

**Design, Setting, and Participants:**

This 2-arm cluster-randomized clinical trial was conducted between January 11, 2021, and May 22, 2022, in hospital care units at Haukeland University Hospital in Bergen, Norway. Statistical analysis was performed from April to May 2025.

**Intervention:**

The intervention group followed a 6-month shift schedule with reduced instances of short daily rest periods, whereas the control group adhered to a 6-month shift schedule maintaining the usual number of short daily rest periods.

**Main Outcomes and Measures:**

Primary analyses followed intention-to-treat principles. The outcome was change in sickness-related absence days and absence spells (ie, each uninterrupted period of ≥1 consecutive sickness-related absence days) over the final 5 months of the intervention (allowing a 1-month stabilization period), compared with the same period in the preceding year and against a control group. The economic returns, measured as the increase in net present value of production from reduced sickness-related absence days due to the intervention, was estimated using a standard cost-benefit formula.

**Results:**

Of 66 hospital units with 811 health care workers (mean [SD] age, 39.8 [12.8] years; 626 of 808 women [77.5%]) in 80% or more full-time positions, 31 units (344 workers) were randomized to the intervention group and 35 units (467 workers) to the control group. The mean (SD) number of short daily rest periods among the intervention group was halved from 18.0 (8.4) during the reference period to 9.1 (6.2) in the intervention period, while the frequency remained unchanged among the control group (reference period, 18.3 [8.3] days; and intervention period, 17.5 [8.4] days). The intervention group showed a significantly smaller increase in sickness-related absence days (incidence rate ratio [IRR], 0.56; 95% CI, 0.41-0.79; *P* < .001) and spells (IRR, 0.73; 95% CI, 0.61-0.86; *P* < .001) compared with the control group. The effect on sickness-related absence days in the intervention units resulted in a positive estimated net economic return of approximately NOK 2 174 620 (USD $213 600) over 5 months.

**Conclusions and Relevance:**

In this randomized clinical trial of health care workers, reducing the frequency of short daily rest periods had positive effects on sickness-related absences and reduced expenses. These findings should guide organizational practices and inform legislative policies to enhance the health of workers by increasing daily rest periods to 11 hours or more between shifts.

**Trial Registration:**

ClinicalTrials.gov Identifier: NCT04693182

## Key Points

**Question:**

Does a shift schedule with fewer short daily rest periods affect sickness-related absence of health care workers compared with a usual schedule, and is there an economic benefit of such a schedule?

**Findings:**

In this cluster-randomized clinical trial that included 66 hospital units and 811 health care workers, a 6-month shift schedule with half the number of short daily rest periods had a positive effect on sickness-related absence days and spells compared with a usual shift schedule. The effect on sickness-related absence days yielded a positive estimated economic return over 5 months.

**Meaning:**

These findings suggests that a shift schedule with fewer short daily rest periods may improve sickness-related absence rates and reduce the associated costs.

## Teaser

This cluster-randomized clinical trial assesses the effect of a shift schedule with fewer short daily rest periods on sickness-related absence among health care workers compared with a usual schedule and evaluates the economic benefit of such a schedule.

## Introduction

Short daily rest periods from work are usually defined as less than 11 hours off between shifts, typically occurring when an evening shift is followed by a morning shift the next day.^1^ In the US, 45% to 50% of retail workers report regularly having short daily rest periods,^2,3,4^ while in the EU, 23% of workers across sectors experience at least 1 short daily rest period per month.^2^ In Scandinavian countries, 63% to 83% of health care employees regularly have short daily rest periods built into their work schedules.^5^ The statistics from the EU and Scandinavian countries represent notable violations of the EU’s Working Time Directive (2003/88/EC, Article 3), which mandates a minimum of 11 consecutive hours of rest within each 24-hour period.^6^ Although the Accreditation Council for Graduate Medical Education requires at least 10 hours off between shifts for US medical residents,^7^ no federal regulations enforce similar standards for other health care professionals,^8^ leading to wide variability across states and institutions.

Short daily rest periods are considered by employees to be among the most unfavorable aspects of working time arrangements.^9^ Such limited rest has been linked with short and poor-quality sleep, sleepiness while awake, fatigue, stress, depressive symptoms, poor work-life balance, and risk of injuries as well as occupational accidents.^1,10,11,12,13,14,15^ Furthermore, short daily rest periods have been associated with increased risk of sickness-related absence.^16^ However, a nonlinear association seems to be present, as a moderate number of short daily rest periods (≤50 per year^17^ or ≤4 per month^18^) may reduce the risk of sickness-related absence, while a high number (>50 per year^17^ or ≥5 per month^18^) is associated with increased risk. Two nonrandomized studies on removing evening-to-day transitions (<11 hours of rest) in health care schedules found improvements in self-reported health, well-being, stress, and mental strain, but no effect on self-reported diseases or sickness-related absence.^19,20^

Sickness-related absence rates are high and increasing in the health care sector,^21^ leading to productivity loss and financial costs for both employers and society.^22^ The World Health Organization projects a shortage of 10 million health care workers by 2030, with global challenges in workforce availability, deployment, and retention.^23^ This projected shortage highlights the need for effective strategies to reduce sickness-related absence and safeguard the health of health care workers. Given the substantial financial burden that sickness-related absence places on hospital budgets as well as the broader societal costs, it is important to evaluate whether reducing short daily rest periods is cost-saving, in line with general recommendations for economic evaluations of organizational interventions.^24^

The aim of this cluster-randomized clinical trial was to assess the effect of a work schedule designed to reduce the occurrence of short daily rest periods compared with a shift schedule that maintained the usual number of short daily rest periods, using objective sickness-related absence data as the primary outcome, and to calculate the potential cost-benefit of the intervention.

## Methods

### Trial Design

The Health-Promoting Work Schedules trial was a 2-arm cluster-randomized clinical trial among rotating-shift health care workers at 66 care units in a Norwegian university hospital. The trial protocol has been published (Supplement 1)^25^ and the study was preregistered with ClinicalTrials.gov (NCT04693182). The study was reported in accordance with the Consolidated Standards of Reporting Trials (CONSORT) reporting guideline for randomized clinical trials.^26^ The study was approved by the Regional Committee for Medical and Health Research Ethics in Western Norway. The enrollment of participants and the collection of hospital register data were exempted from individual consent, as detailed in the trial protocol; a key factor for the exemption was that the intervention involved implementing a work schedule that was expected to benefit employees’ health and safety without causing harm (Supplement 1).^25^

All 24-hour staffed care units at the hospital were considered for eligibility. Unit-level inclusion criteria were having employees who (1) were health care workers (not including physicians) on regular and/or irregular rotating shifts, (2) regularly had short daily rest periods in their shift schedule, and (3) had a new shift rotation year starting in the first half of 2021. Physicians were excluded due to different shift schedules and compensation schemes. Unit-level exclusion criteria were (1) recent or upcoming major organizational changes during the intervention period or the corresponding 6-month reference period from the previous year that could confound trial results or (2) strong opposition to participating in the trial from the unit’s manager or a substantial number of employees. In addition, intensive care units treating patients with COVID-19 were excluded due to the pandemic and the unique, stressful working conditions (eTable 1 in Supplement 2). Individual-level inclusion criteria were to have payroll data showing actual working hours corresponding to 80% or more of a full-time position during the respective unit’s intervention period and the reference period in the preceding year. These data were calculated based on total hours worked during each 6-month period, with 834.25 hours representing full-time work for 6 months, which is the norm for health care workers in Norway.

### Randomization and Intervention

Between August 19 and September 10, 2020, hospital care units (clusters) were randomized on a 1:1 basis to either a 6-month shift schedule with minimal short daily rest periods (intervention) or a schedule maintaining the usual number of short daily rest periods (control). To ensure that units had comparable numbers of short daily rest periods, they were stratified by medical function into 10 subgroups (2-19 units each). In strata with an odd number of units, the extra unit was randomly assigned to 1 of the 2 conditions. Randomization was conducted using a computer-generated sequence. Given the nature of the intervention, blinding health care workers or unit managers to a condition was not possible. Initial analyses were conducted by a statistician (S.A.L.) blinded to group allocation. Subsidiary and cost-benefit analyses were conducted without blinding.

After randomization in September 2020, units planned their 6-month shift schedules according to their assigned condition. Intervention units aimed to eliminate short daily rest periods in the schedules, but complete removal proved infeasible due to staffing constraints, absences, and last-minute shift changes. Human resources supported managers with guidance and sample schedules (eTable 2 in Supplement 2). Intervention units could implement the 6-month intervention schedule in either half of the shift rotation year. Control units were matched by size and medical function to ensure comparable workload and work demands and to align observation periods (ie, reference and intervention periods), minimizing seasonal effects on sickness-related absence. They maintained usual shift scheduling with no changes in the number of short daily rest periods. The first intervention units implemented their schedules on January 11, 2021, with the final unit completing the period on May 22, 2022.

### Outcomes

Data were retrieved from the hospital’s local records at the individual level. Demographic information on sex (female and male), age (2021 – birth year), and seniority (date of intervention start – date of employment at the hospital) was retrieved from the employee information records. Information on working hours (payroll data) and sickness-related absence (absence data) was retrieved for the intervention period and the corresponding period the year before (reference period) (ie, if the intervention period lasted from February 1 to July 31, 2021, then data from February 1 to July 31, 2020, constituted the reference period).

Data on the primary outcome, sickness-related absence, included all self-certified and medically certified sickness-related absence caused by the employee’s own illness, whereas absences due to caregiving responsibilities (eg, childcare) or mandatory quarantine related to COVID-19 without illness were excluded. The total number of sickness-related absence days and absence spells (ie, each uninterrupted period of ≥1 consecutive sickness-related absence days) was compiled for the final 5 months of the intervention period and the corresponding reference period, allowing a 1-month stabilization period to the new schedule.

Data on work hours included information on shift records for all included health care workers in the involved units during the intervention and reference periods, detailing the date and start and end times of all completed shifts. Shifts separated by 1 hour or less were merged into one longer shift, and only active shifts were included (on-call shifts were excluded). Shift characteristics were classified according to previous work on payroll data into day, evening, or night shifts^5^ and consecutive periods of evening and night shifts were identified. Based on established literature, shift schedules were categorized as permanent, 2-shift, or 3-shift schedules.^5,27^ Short daily rest periods were defined as transitions between shifts with less than 11 hours off.^1^ The number of short daily rest periods was counted, and the time between shifts in these changeovers was calculated. Adherence to the intervention was indicated by any reduction in the number of short daily rest periods from the reference period to the intervention period.^25^

Toward the end of the intervention period, employees in both groups were invited to provide feedback on potential unwanted negative events or effects by responding to a set of trial-specific questions (the trial protocol is provided in Supplement 1).^25^ Employees were also encouraged to report any adverse effects of the new shift schedules directly to the research team.

### Sample Size

The trial aimed to include all hospital units and health care workers who met the prespecified inclusion and exclusion criteria.^25^ Initial power analyses indicated that 2028 health care workers were needed to detect a difference in sickness-related absence days ranging from 0.9 to 1.25,^16^ assuming an intraclass correlation coefficient of 0.1 across 76 clusters, each with a mean (SD) of 52 (0) employees.^25^ Before applying any criteria, the available sample consisted of 3393 employees, with a mean full-time equivalent of just over 60% during both reference and intervention periods. According to the protocol, the primary analysis was planned for employees working equivalent to 80% or more of a full-time position.^25^ This reduced the sample to 811 health care workers, leaving the study underpowered to detect the expected effect on sickness-related absence. To improve power and assess variability, additional analyses including those working 50% or more of a full-time position (n = 1764) were preregistered.

### Statistical Analysis

Statistical analysis was performed from April to May 2025. Intervention effects were assessed using multilevel negative binomial regression models comparing sickness-related absence days and spells between intervention and control units over time, reported as incidence rate ratios (IRRs) with 95% CIs. This model accounted for the count nature of the outcomes and clustering within hospital units (random effects), with standard errors estimated via maximum likelihood. Fixed effects included randomization group (reference, control), time (reference, reference period), and their interaction (difference-in-difference). Primary analyses followed intention-to-treat principles and included health care workers working 80% or more of a full-time position, with secondary analyses including those working 50% or more of a full-time position. There were no missing data on the primary outcomes, as sickness-related absence information was obtained from hospital records. All analyses were conducted in R, version 4.4.1 (R Project for Statistical Computing).^28^ For presentation purposes, population-averaged predicted means on the count scale were reported using the emmeans package, integrating over the estimated random-effects distribution (adaptive Gauss-Hermite quadrature) and back-transforming the linear predictor with a bias-adjustment of ½ σ^2^.

The trial was conducted under naturalistic conditions, allowing employees to work across both intervention and control units due to split roles or staffing needs. This included 1 employee working 80% or more of a full-time position and 9 employees working 50% or more of a full-time position of a full-time position who were classified based on their primary unit during the intervention. Given the small number of employees and limited effect on results, no sensitivity analyses were conducted to assess their exclusion. However, post hoc sensitivity analyses were conducted adjusting for baseline values of sickness-related absence, using negative binomial models with robust standard errors and adjusting for stratification (eTable 3 in Supplement 2).

Potential economic returns for society were calculated using the following standard cost-benefit formula based on the human capital approach^29,30,31^:


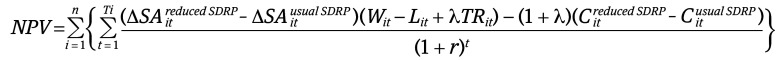
.

This formula estimated the potential net present value of increased production from reduced sickness-related absence days. Neither health care workers nor the employer reported additional costs from a reduction in short daily rest periods; therefore, net cost increases were excluded from the calculations. Calculations were limited to 5 months and 1 (first) year, assuming they worked a mean of 90% of a full-time position annually. Additional analyses including workers working 50% or more of a full-time position were conducted (eTable 5 in Supplement 2). Estimated returns are presented for the intervention group, all trial participants, and all rotating shift workers at the hospital. Additional benefits will accumulate over time if the intervention’s effect is sustained.

To examine potential differences between groups in self-reported potential unwanted negative events or effects, we fitted a cluster-adjusted cumulative-logit mixed model (R package ordinal, clmm), with shift-schedule group (intervention vs control) as a fixed effect and unit (clusters) as a random intercept for each of the items. Odds ratios (ORs) and Wald *P* values were extracted for the group term, and a Bonferroni correction (α/18) was applied to control the family-wise error rate (α ≈ 0.0028). All *P* values were from 2-sided tests and results were deemed statistically significant at *P* < .05.

## Results

Of 66 hospital units with 811 health care workers (mean [SD] age, 39.8 [12.8] years; 626 of 808 women [77.5%] and 182 of 808 men [22.5%]) working 80% or more of a full-time position, 31 units (344 workers) were randomized to the intervention group and 35 units (467 workers) to the control group (Figure). Apart from the proportion of men (130 of 466 [27.9%] in the control group and 52 of 342 [15.2%] in the intervention group), the outset characteristics were evenly distributed between groups (Table 1). During the reference period, 243 of 444 health care workers (54.7%) had at least 1 sickness-related absence day in the last 5 months of the period, with 57.3% in the intervention group (197 of 344) and 52.9% in the control group (247 of 467). Among the intervention group, the mean (SD) number of short daily rest periods was reduced from 18.0 (8.4) during the reference period to 9.1 (6.2) during the intervention period (Table 2). In contrast, the mean (SD) number of short daily rest periods remained comparatively unchanged among the control group from the reference period (18.3 [8.3]) to the intervention period (17.5 [8.4]). An exploratory mixed-effects analysis confirmed that the intervention group significantly reduced the mean number of short daily rest periods by 44% compared with the control group (IRR, 0.56; 95% CI, 0.52-0.61; *P* < .001). In occurrences of short daily rest periods, the time between shifts was consistently around 9 hours across time and groups. There were no other notable differences in shift characteristics across the measurement points or groups.

**Figure.**
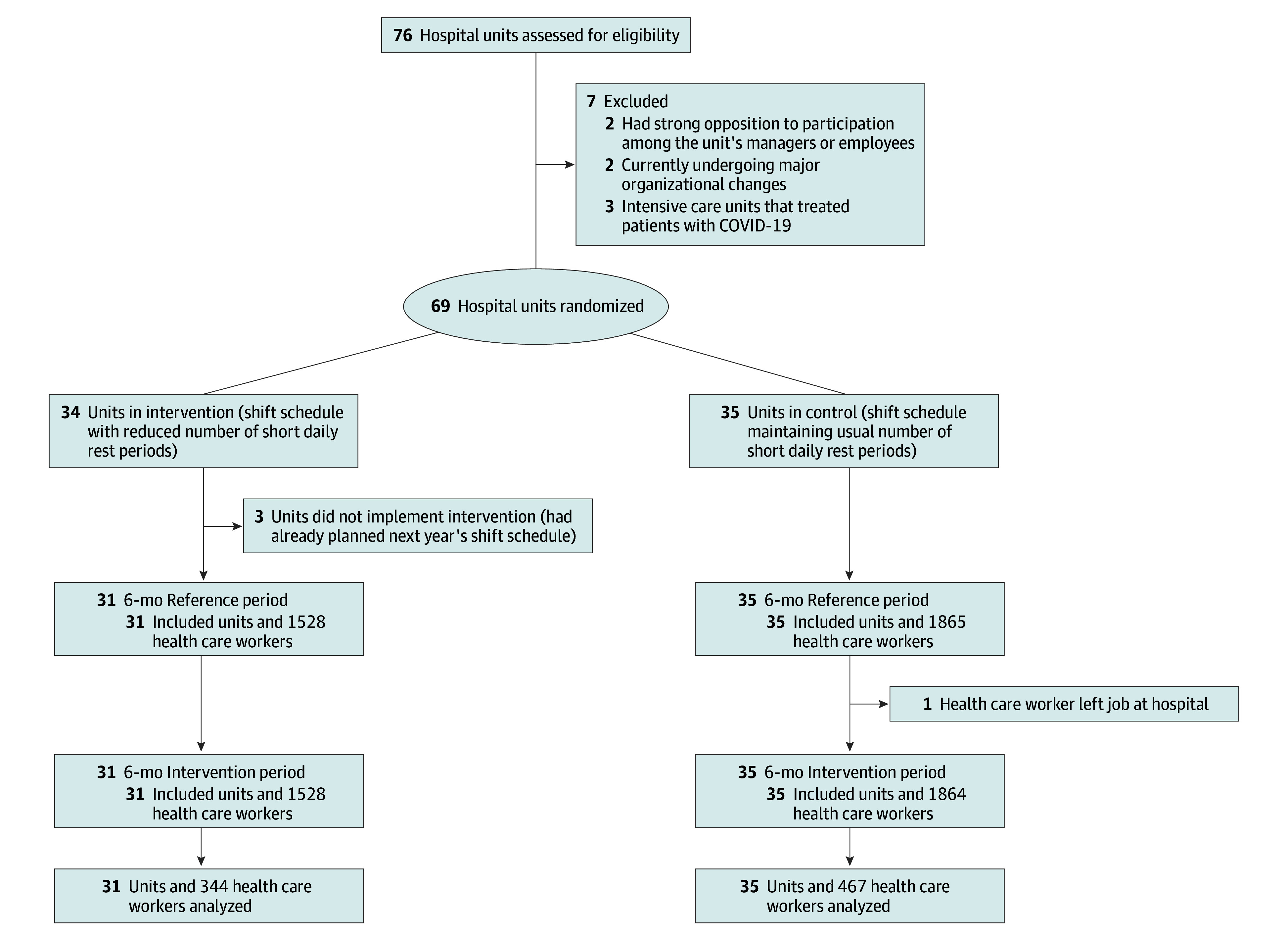
Study Flow Diagram

**Table 1.** Characteristics of Hospital Units (Clusters) and Health Care Workers

Characteristic	Shift schedule		
	Reduced No. of short daily rest periods (n = 344)	Usual No. of short daily rest periods (n = 467)	Total (N = 811)
Hospital units			
No. of clusters	31	35	66
Cluster size, mean (SD) , no. of health care workers	11.1 (5.6)	13.3 (6.14)	12.3 (5.9)
Health care workers			
Intervention start time			
First quarter	210/344 (61.0)	286/467 (61.2)	496/811 (61.2)
Third quarter	134/344 (39.0)	181/467 (38.8)	315/811 (38.8)
Age, mean (SD), y^a^	41.1 (13.1)	38.8 (12.4)	39.8 (12.8)
Sex			
Female	290/342 (84.8)	336/466 (72.1)	626/808 (77.5)
Male	52/342 (15.2)	130/466 (27.9)	182/808 (22.5)
Occupational group			
Nurses	244/300 (81.3)	304/403 (75.4)	548/703 (78.0)
Health care assistants	18/300 (6.0)	56/403 (13.9)	74/703 (10.5)
Other health care personnel	38/300 (12.7)	43/403 (10.7)	81/703 (11.5)
Seniority, mean (SD), y^b^^,^^c^	12.9 (9.8)	10.6 (8.4)	11.6 (9.1)
% Of full-time position worked, mean (SD)^d^	94.3 (9.0)	95.2 (9.5)	94.8 (9.3)

^a^ Unless indicated otherwise, data are presented as No./total No. (%) of health care workers. Due to missing data from the hospital’s employee information register, data on age were available for 806 health care workers.

^b^ Refers to seniority that provides a basis for salary.

^c^ Due to missing data from the hospital’s employee information register, data on seniority were available for 786 health care workers.

^d^ Percentage of full-time position worked, based on total number of hours worked during the 6-month reference period.

**Table 2.** Shift Schedule Characteristics Among Health Care Workers

Characteristic	Shift schedule			
	Reduced No. of short daily rest periods (n = 334)		Usual No. of short daily rest periods (n = 467)	
	Reference period	Intervention period	Reference period	Intervention period
Units	31	31	35	35
Type of shift schedule				
Fixed day shifts	49 (14.2)	51 (14.8)	44 (9.4)	46 (9.9)
Fixed evening shifts	0	0	0	0
Fixed night shifts	17 (4.9)	17 (4.9)	36 (7.7)	39 (8.4)
Two-shift schedule: day and evening	105 (30.5)	103 (29.9)	149 (31.9)	148 (31.7)
Two-shift schedule: day and night	7 (2.0)	8 (2.3)	3 (0.6)	3 (0.6)
Two-shift schedule: evening and night	1 (0.3)	0	5 (1.1)	7 (1.5)
Three-shift schedule: day, evening, and night	165 (48.0)	165 (48.0)	230 (49.3)	224 (48.0)
No. of shifts, mean (SD)				
Day shifts	56.2 (18.2)	57.3 (19.3)	54.2 (18.7)	53.7 (19.3)
Evening shifts	30.7 (12.6)	29.1 (13.4)	33.2 (13.1)	32.7 (13.9)
Night shifts	11.6 (13.0)	11.9 (14.3)	11.8 (13.9)	12.4 (16.1)
Periods with consecutive shifts, mean (SD)				
≥4 Consecutive night shifts	0.3 (1.2)	0.3 (1.1)	0.3 (1.3)	0.5 (1.8)
≥4 Consecutive evening shifts	0.1 (0.4)	0.3 (0.7)	0.2 (0.8)	0.2 (0.8)
≥2 Consecutive evening shifts	6.0 (4.6)	7.0 (5.1)	6.6 (4.9)	6.9 (4.9)
Short daily rest periods, mean (SD)^a^				
No. of occurrences^a^	18.0 (8.4)	9.1 (6.2)	18.3 (8.3)	17.5 (8.4)
Time between shifts, h:min^a^	09:04 (00:41)	09:04 (00:32)	09:08 (00:23)	09:04 (00:31)

^a^ Unless indicated otherwise, data are presented as No. (%) of health care workers. Short daily rest period refers to less than 11 hours off between 2 consecutive shifts.

Although both groups displayed an increase in mean sickness-related absence days from the reference period to the intervention period, the intervention group had a significantly lower increase in sickness-related absence days (IRR, 0.56; 95% CI, 0.41-0.79; *P* < .001) and spells (IRR, 0.73; 95% CI, 0.61-0.86; *P* < .001), compared with the control group (Table 3). Sensitivity analyses also yielded similar results, confirming the robustness of the main findings (eTable 3 in Supplement 2).

**Table 3.** Results From Intention-to-Treat Analysis on Sickness-Related Absence During the Last 5 Months of the Intervention

Characteristic	Shift schedule				Intervention effect	
	Reduced No. of short daily rest periods		Usual No. of short daily rest periods			
	No.	Mean (SE)^a^	No.	Mean (SE)^a^	IRR (95% CI)	*P* value
**Sickness-related absence days^b^**						
Reference period	334	3.9 (0.6)	467	2.6 (0.4)	1 [Reference]	NA
Intervention period	334	4.5 (0.6)	467	5.4 (0.7)	0.56 (0.41-0.79)	<.001
**Sickness-related absence spells^c^**						
Reference period	334	1.1 (0.1)	467	1.0 (0.1)	1 [Reference]	NA
Intervention period	334	1.3 (0.1)	467	1.6 (0.1)	0.73 (0.61-0.86)	<.001

Abbreviations: IRR, incidence rate ratio; NA, not applicable.

^a^ Predicted marginal (population-averaged) means from the mixed-effects negative-binomial model; random intercept variances for unit and employee were integrated out before back-transformation.

^b^ Sickness-related absence during the last 5 months of the reference period and last 5 months of intervention period.

^c^ Sickness-related absence spells (ie, each uninterrupted period of ≥1 consecutive sickness-related absence days) during the last 5 months of the reference period and last 5 months of the intervention period.

Based on the attenuated increase in sickness-related absence days in the intervention group relative to the control group, the net present value of implementing the intervention over 5 months for the 344 health care workers in the intervention group was estimated at NOK 2 174 620 (USD $213 600; adjusted calculation; Table 4). Extending the intervention to all 811 trial health care workers over a full year, assuming the observed effect was sustained, yielded an estimated net present value of NOK 12 304 303 (USD $1 208 579; adjusted calculation). Applying the same effect to all rotating shift workers working 80% or more of a full-time position at the hospital, excluding physicians (4260 health care workers, per January 2024), was estimated to generate an annual net present value of NOK 64 631 729 (USD $6 348 394; adjusted calculation). USD values were calculated using the Central Bank of Norway’s (Norges Bank) official exchange rate on August 18, 2025 (1 USD = 10.1808 NOK; 1 NOK = 0.0982 USD).

**Table 4.** Elements in the Calculation of the NPV of Economic Returns for Society From a Shift Schedule With Reduced Number of Short Daily Rest Periods^a^

Element	≥80% of a Full-time position^b^	
	100%	90%^c^
Annual gross wage (mean, health care workers at the hospital, 2023), NOK	630 000	567 000
Payroll tax (14%)	88 200	79 380
Mandatory pension plan (2%)	12 600	11 340
Payroll tax (14%) on pension plan	1764	1588
Wi: value of production	732 564	659 308
Sickness benefits net of income tax (Gross wage × 0.66)	415 800	374 220
Income tax (34%) and payroll tax (14%)	304 164	273 748
TRi: reduction in transfers if working	719 964	647 968
λ: Dead weight loss from taxation (20%)	143 993	129 594
Li: Value of leisure when on sickness-related absence	120 582	108 524
r: Discount rate	0,04	0.04
TE^d^	−5.16	−5.16
Extra costs from reduced SDRP shift schedule	0	0
NPV per person per year not on sickness-related absence	726 899	654 209
NPV per person per day not on sickness-related absence (222.5 workdays per year)	3267	2940
NPV from treatment of the treated (TE 5 mo × value per day × i)	2 416 244	2 174 620
Annual NPV if treatment of all included in the project (TE 12 mo × value per day × No. of health care workers)	13 671 448	12 304 303
Annual NPV if implemented in Helse Bergen (No. working ≥80% of a full-time position = 4260 health care workers)^e^	71 813 032	64 631 729

Abbreviations: i, No. treated; NOK, Norwegian kroner; NPV, net present value; SDRP, short daily rest periods; TE, treatment effect; t, periods with TE.

^a^ The calculations follow the principles recommended by the Norwegian Ministry of Finance (R-109-2021).

^b^ Based on treatment effects and numbers of treated, numbers of participants who worked 80% or more of a full-time position.

^c^ Assuming workers, over the calendar year, worked on average 90% of a full-time position.

^d^ TE refers to the annual effect (in days) of implementing a shift schedule with a reduced number of short daily rest periods.

^e^ All workers refer to workers in all occupations, except physicians, in rotating shift schedules at Haukeland University hospital per January 2024.

Similar adherence patterns were observed in the secondary sample of health care workers working 50% or more of a full-time position, with the intervention group reducing the mean (SD) number of short daily rest periods from 13.2 (9.2) to 6.8 (6.1), while the number remained stable in the control group (13.0 [9.5] to 12.5 [9.5]). Likewise, the intervention effects in this sample mirrored those seen in the sample of health care workers working 80% or more of a full-time position (eTable 4 in Supplement 2), and also yielded a positive estimated net present value (eTable 5 in Supplement 2).

A total of 600 health care workers (intervention; 316; control, 284) responded to the trial-specific questions regarding potential unwanted negative events or effects (eTable 6 in Supplement 2). Compared with the control group, the intervention group had lower odds of reporting more sleep problems (OR, 0.57; 95% CI, 0.40-0.81; *P* = .002), but higher odds of experiencing their shift schedule as more unfavorable (OR, 2.13; 95% CI, 1.55-2.92; *P* < .001), less flexibility to swap shifts (OR, 2.58; 95% CI, 1.62-4.11; *P* < .001), and worse continuity of patient care (OR, 2.62; 95% CI, 1.55-4.45; *P* < .001) (eTable 7 in Supplement 2). Most respondents in both groups reported “not at all” in response to the list of unwanted events or effects, and no adverse events were reported to the research team during the trial.

## Discussion

This cluster-randomized clinical trial found that halving the number of short daily rest periods in the work schedule had positive effects on sickness-related absence among health care workers working equivalent to 80% or more of a full-time position, compared with a control group. The results showed an estimated economic return for society of NOK 2 174 620 (USD $213 600) over 5 months, based on calculations for the effect on the 344 health care workers in the intervention group (adjusted calculations).

The findings are consistent with previous observational studies showing that short daily rest periods are negatively associated with sickness-related absence rates^16,17,19^ and that reducing their frequency is associated with beneficial effects.^11^ Although a small (n = 75) nonrandomized study found no change in self-reported sickness-related absence after reducing short daily rest periods,^19^ our results are consistent with those of a recent quasi-experimental study reporting a smaller increase in sickness-related absence in hospital units required to reduce short daily rest periods under a national reform.^32^ Using objective payroll data in a large cluster-randomized design, our study strengthens the evidence for a causal link between reduced short daily rest periods and improved sickness-related absence.

Although the intervention halved the frequency of short daily rest periods, it remains unclear whether complete elimination of short daily rest periods would yield additional benefits. Some studies suggest a nonlinear association between short daily rest periods and sickness-related absence, indicating that maintaining a few short changeovers may be beneficial.^17,18^ These short rest periods may offer scheduling advantages, such as compressed work weeks and longer consecutive time off, which some employees highly value, and may also support continuity in work processes and information transfer.^33^ Some employees prefer such schedules,^20^ possibly because practical advantages may outweigh associated discomfort and risks. This was reflected in our questionnaire findings, where the intervention was perceived as less flexible, more unfavorable, and detrimental for patient care continuity, despite fewer reported sleep problems. A qualitative study similarly noted that health care workers recognize these tradeoffs.^34^ Together, these perspectives suggests that the optimal level of short daily rest periods may not depend only on health outcomes but also on employee preferences and operational needs.

Disturbed sleep^15,35^ and inadequate recovery^36^ likely explain the negative effects of short daily rest periods. Short daily rest periods have been linked with difficulties falling asleep^37^ and unwinding from work,^38^ as well as shorter sleep duration.^39^ Epidemiologic studies show that insufficient sleep is associated with increased risk of workplace accidents, depression, type 2 diabetes, stroke, coronary heart disease, and inflammatory markers,^35^ which may help account for the effects observed in our trial. Previously reported findings from the current trial showed positive effects on symptoms of insomnia and daytime sleepiness.^15^

### Strengths and Limitations

This study has some strengths. The use of register data both to assess trial adherence and measure sickness-related absence eliminated subjectivity-related biases (eg, recall or measurement bias) and ensured no missing data. Unintended changes in the shift schedule that might have come with the intervention were assessed (eg, more consecutive evening shifts), but no such changes were detected (Table 2). As for the cost-benefit analysis, one may argue in favor of using the friction cost (ie, a method that estimates productivity losses based on the time it takes to replace and absent worker, assuming production is eventually restored) rather than the human capital approach for calculating the economic returns for society.^40,41^ Production is often maintained in cases of sickness-related absence in health care due to substitutes, but because there is a shortage of health care workers, the substitutes have alternative employment opportunities elsewhere, which ultimately affects production.

Although the trial enrolled only 811 participants in the sample working 80% or more of a full-time position instead of the planned 2028 participants, the significant intervention effect suggests a larger-than-anticipated effect size.^25^ Still, the smaller sample size raises the risk of type II error. However, the absence of missing data and the robustness of the findings across sensitivity analyses strengthen the findings and may mitigate concerns about the sample size. Furthermore, given that part-time work is common among Norwegian health care workers,^42,43^ the replication of effects in the sample working 50% or more of a full-time position strengthens the generalizability and relevance of the findings.

This study also has some limitations. The trial’s naturalistic setting made it challenging to estimate the number of health care workers in the trial and fully account for any baseline differences (ie, reference period) between groups. The latter was also partly caused by the cluster design. The mean number of sickness-related absence days was higher in the intervention group than in the control group across all months in the reference period (eFigure in Supplement 2). Although this pattern was stable over the observed 6 months, access to data from a longer preintervention time frame would have enabled a more robust evaluation of underlying trends and strengthened the attribution of observed changes to the intervention. Sex distribution differed slightly between the trial groups; however, nonhypothesized differences between groups were not adjusted for in the analyses.^26^

## Conclusions

In this randomized clinical trial of health care workers, reducing the number of short daily rest periods for health care workers improved workforce health and reduced costs. This underscores the need for changes in workplace practices and policy adjustments to reduce the number of short daily rest periods between shifts. However, given the practical advantages of allowing short daily rest periods and that some employees may prefer these schedules for personal or practical reasons, these findings should not justify a complete ban or removal of such schedules. Nonetheless, the results support a more restrictive use of short daily rest periods in shift schedules, emphasizing the need for further research and a balanced approach to scheduling.

## Notes

Supplement 1.
Trial Protocol

Supplement 2. **eFigure.** Observed Mean Sickness Absence Days per Month During the 6-Month Reference and Intervention Periods, Stratified by Randomization Group for Healthcare Workers Working Equivalent to ≥ 80% of a Full-Time Position **eTable 1.** Table Outlining the Changes to the Study During the Project Period Due to the COVID-19 Pandemic **eTable 2.** Examples of a Two-Week Cycle of Rotating Shift Work With and Without Short Daily Rest Periods (<11 Hours off Between Two Consecutive Shifts) **eAppendix 1.** Sensitivity Analyses **eTable 3.** Results From the Sensitivity Analyses Where the Intervention Effect Was Adjusted for the Baseline (i.e., Reference Period) Value of Sickness Absence and Analyses Also Using Flat Negative Binomial Models With Robust Standard Errors and Baseline Adjustment in Healthcare Workers Who Worked Equivalent to ≥80% of a Full-Time Position (N = 811) **eAppendix 2.** ITT and Sensitivity Analyses in the ≥50% Position Sample **eTable 4.** Results From Intention-to-Treat and Sensitivity Analyses on Primary Outcomes on Healthcare Workers Who Worked Equivalent to ≥50% of a Full-Time Position (n=1,764) **eAppendix 3.** Supplementary Cost-Benefit Analysis **eTable 5.** Elements in the Calculation of the Net Present Value (NPV) of Economic Returns for the Society From a Shift Schedule With a Reduced Number of Short Daily Rest Periods for Healthcare Workers Working Equivalent to ≥50% of a Full-Time Position **eTable 6.** Total Number of Responses and Frequency Distribution to Questions About Possible Unwanted Negative Events or Effects as a Result of the Shift Schedule Over the Last Few Months. Responses Were Given at the End of the Six-Month Intervention Period (n=600) **eTable 7.** Cluster-Adjusted Ordinal-Logistic Regression Results for Self-Reported Questions About Possible Unwanted Negative Events or Effects as a Result of the Shift Schedule the Last Few Months at Six-Month Follow-up (Intervention vs Control)

Supplement 3.
Data Sharing Statement
