# Supplementary material for: Shift Schedule With Fewer Short Daily Rest Periods and Sickness Absence Among Health Care Workers: A Cluster Randomized Clinical Trial
Source: JAMA Netw Open. 2025 Sep 15;8(9):e2531568. doi: 10.1001/jamanetworkopen.2025.31568 (PMC12439055; doi:10.1001/jamanetworkopen.2025.31568)
Supplement: Supplement 1. — Trial Protocol [file jamanetwopen-e2531568-s001.pdf]

Towards a sustainable work force in the healthcare sector for the 21st century: Health-promoting Work Schedules (HeWoS)

1 Excellence

1.1 State of the art, knowledge needs and project objectives

Within the healthcare sector in Europe, about one third of the workforce consists of shift workers.[1] Labour needs lead to an increased pressure on the workers and force them to be engaged in longer work sessions and more shift work. This comes at a cost of high absence due to sickness[2] and high turnover rate[3,4].The need for healthcare workers is in turn expected to increase[2]. Thus, is imperative to identify health-promoting work schedules to maintain the health and well-being of healthcare workers, and ensure that they are able to remain in the profession until natural age retirement.[5] **HeWoS aims to map effective schedule for healthcare workers to ensure a sustainable work force in the healthcare sector for the future. The project will collaborate with the large health enterprises in Norway and will conduct large-scale studies and comparisons to the registries in Denmark and Finland.**

Shift work, health and work-related accidents. Sleep, wakefulness and several other bodily processes oscillate according to a 24-hour rhythm (circadian rhythm). These rhythms are governed by the superchiasmatic nuclei (SCN), which normally has an endogenous period that is somewhat longer than 24 hours.[6] The SCN is typically entrained to a 24-hour rhythm by time givers such as daylight, physical activity, meals etc.[7] Circadian rhythmicity is evident in the core body temperature, which typically falls in the evening and continues to drop to its lowest point (nadir) in the early morning hours, where after it rises.[8] The circadian rhythm strongly affects human performance, which is poorer during nighttime compared to daytime. This is reflected in reduced productivity and increased risk of accidents during night shifts.[9] In addition, shift and night work has been found to impact several health parameters, like sleep problems, cardiovascular disease, metabolic syndrome, and cancer, among others.[9]

Disturbed sleep is the most common health complaint among shift workers. Shift work disorder (SWD) is a circadian rhythm sleep disorder characterized by excessive sleepiness and complaints of insomnia related to the work schedule.[10] Among nurses, the prevalence of SWD has been reported from 24%[11] and up to 44%.[12] Nurses with SWD report more headaches and migraine[13] and Restless Legs[14] compared to nurses who do not have SWD. Shift work related sleep problems have also been shown to predict exclusion from work and turnover.[15]

The evidence linking night shifts to sleep problems and other health complaints is strong.[9] However, resent studies have indicated that on some parameters there may be other schedule characteristics that evoke as much, or perhaps even more, discomfort for the workers as that with night shifts. This pertains to so called quick returns (QRs) which occur in rotating shift schedules, primarily in changeovers from evening to day/morning shifts and is defined as rest time of less than 11 hours between shifts.[16] QRs are common among some occupational groups, where one study from our group indicated that 81% of nurses reported having QRs in their schedule, with a mean of 33 QRs per year.[17] QRs more often occur in the work schedule of Norwegian nurses as compared to Danish and Finnish nurses.[18] One study on workers from a wide range of occupations showed that 28% of workers that had QRs reported that this was a problem in their schedule, whereas only 12% of those that had night work reported that this was a problem in their schedule.[19] Studies suggest that workers report relatively more sleep related disturbances[17,20] and have more sickness absence with QRs[16] than night shifts. QRs have also been associated with short sleep duration, sleepiness, feelings of exhaustion, fatigue, work to family interference, gastrointestinal problems, and work related accidents.[21-24] Nurses with many QRs also reported less satisfaction with their working time arrangements,[21] which is a factor that is important for turnover and the ability to remain in the profession. So far, these studies have exclusively established an association between QRs and negative outcomes, while it is necessary to establish the causal relationship between QRs and negative outcomes before recommendations to abolish QRs from the shift schedule can be issued. QRs are scarcely studied compared to night shifts, and the results referred to above point to a need to further investigate possible negative consequences of QRs.

Shift work and sickness absence. Absence from work due to sickness is commonly used as a surrogate measure of the workers' health.[25] Norway has the highest long-term sickness absence rates among the Nordic countries.[26] Comparison between countries can be challenging due to variations across countries in the information contained in the official registers. However, more detailed records of the employee's sickness absence are kept by the employers, and researchers in Denmark and Finland have established a national shift work register based on such payroll data.[27] This enables a more accurate comparison of shift related sickness absence across countries. It is important to establish such a register in Norway as well, which will contribute to a better comparison across countries and a better understanding of the underlying causes of absence due to sickness. In Norway, healthcare workers have the highest sickness absence rates

compared to other professions, amounting to 8.7% in the last quarter of 2018.[2] Still, little is known about the potential role of shift work exposure in these absence rates. Research on shift work and sickness absence is scarce and characterized by many contradictory findings.[28] The latter is likely due to methodological shortcomings and differences across studies. For example, the comparability of the reference groups used across studies has been brought into question. In addition, previous sickness absence is rarely considered in predictor models,[29] which can lead to an inflation of findings and conclusions. Most studies have also been based on cross-sectional designs and there is a need for more longitudinal studies and experimental designs that can establish cause and effect. A major limitation in previous research has been imprecise measurement of exposure to shift work (typically based on subjective reports), which render much of the existing research on the association between shift work and negative health outcomes uncertain. It is only recently that researchers have started to utilize objective payroll data to measure shift work exposure, something that also allows us to identify exposure to shift work characteristics at a level of detail not previously possible.[16,29] For example, by using objective payroll data, we found that nurses on average had three QRs per month, which was associated with 21% more sickness absence days the following month, compared to those who had no QRs in their schedule.[16,27,30] However, this was an observational study that cannot say anything certain about causality. So far, the results concerning the association between sickness absence and shift work are equivocal and effect studies on this topic are therefore needed.

Shift work and individual tolerance Some people are able to work shifts without experiencing negative consequences and seem to have a better tolerance for shift work than others. For example, studies in this realm have shown that eveningness (preference for evening as opposed to morning activities) is positively associated with shift work tolerance, especially night work[31]. In addition, individuals who are flexible in terms of times for sleep and work tend to show better tolerance to shift work,[32] and especially to rotating shift work than their counterparts.[33] Other individual characteristics that have been linked to shift work tolerance are low scores on languidity (i.e. difficulties overcoming drowsiness upon losing sleep),[33] low scores on neuroticism and low scores on extraversion,[32] More recent studies on this topic have identified specific genotypes relates to shift work tolerance [34-37]and more studies on this topic are warranted.

Shift work and light exposure. The immediate effects of night work are often demonstrated in terms of impairment in the individuals' alertness and performance on duty. Moving from day-activity to night-activity produces a mismatch between the internal biological timing of alertness and the work schedule. This has major implications for sleep and performance. Consistently, there are reports of impaired productivity[38] and increased risk of accidents[39] during night shifts compared to day shifts. Light is the strongest time-giver for the circadian rhythm.[40] We therefore expect that exposure to daylight after night shifts may affect the workers ability to adapt to shift work. This topic has not been explored specifically in healthcare workers, however, research on offshore workers has shown that light may be used to impede/facilitate adaptation.[41,42] In Norway, the exposure to daylight varies greatly depending on location (latitude) and time of year. This provides a unique opportunity to compare and explore how different light conditions affect adaptation to shift work. We will use this opportunity to compare the predictive effect of working time arrangement on absence due to sickness, adjusting for different daylight exposures (shift workers in different parts of Norway). Shift work simulation studies on the effects of QR have so far not been conducted, despite the fact that such studies are well suited to objectively assess the performance impairment associated with QR, potential moderating factors (e.g. genotypes and personality) and work environment interventions (like bright light) that may counteract the negative effects of QR in terms of performance impairment and sleepiness.

Main objective and sub-objectives. To establish a knowledge base on health-promoting work schedule among healthcare workers and to influence productive and sustainable work force in the healthcare sector in the future. a) To establish a national register for healthcare workers in the public sector based on payroll data. b) To examine effects of abolishing QRs from the work schedule on sickness absence and turnover intention. c) To identify individual differences and specific genotypes related to QR tolerance. d) To establish the effects of QRs on work performance and investigate if light differences can counteract the negative effect of QR. e) To evaluate if a low-threshold intervention (a self-help book) can improve health and reduce turnover among those who struggle with SWD.

1.2 Novelty and ambition

Novel methodology in shift work research. It is not clear which shift systems best preserves the workers' health, and how workers can better cope with shift work. Establishing a large-scale registry allows identification of smaller subgroups that naturally have changed their work schedule over the available record period. Clever use of registry data permits to mimic true experiments in terms of establishing causality and

evaluate the effects of different work schedule exposures on sick leave. This approach allows us to cost-effectively study the entire population of shift workers within the healthcare sector. The use of registry data on exposure and outcome reduces the burden of participating for nurses and hospital departments. The payroll and sickness absence records comprise multi-state data. Multi-state analyses allow for incorporation of repeated shifts between states (e.g. sick leave, work etc.) and can take into account time dependent and shifting exposures as shown in previous publications.[43,44] A national registry will include workers from the southern- and northern parts of Norway, where seasonal differences in daylight offer a unique opportunity to study how daylight exposure affects adaptation to shift work. **HeWoS will be the first shift work register with the opportunity to examine the moderating effect of seasonal fluctuations in light exposure on the association between shift work and sickness absence.** A comparison of the detailed sickness absence records between the Nordic countries (that includes data from the first day of absence) may improve our understanding of the underlying causes of sickness absence in these countries. In collaboration with our colleagues in Denmark and Finland, that have established identical registries, we will agree on the working hour terminology and shift classifications which in its entirety will contribute to a significant improvement in the quality of shift work research in the world.

Causality. QRs are frequent shift characteristics for healthcare workers and studies suggests it is associated with negative health outcomes. **HeWoS will be the first study using an RCT-design to establish the effect of QRs on sickness absence, sleep, health and work-related accidents.** Simulation studies investigating the effects of QRs have not previously been conducted. Nor has there been studies aimed at identifying factors that can moderate the negative effects of QR (i.e., personality, genotypes) and whether light can be used as a countermeasure to the negative effects of QRs. **HeWoS will be the first shift work simulation study to investigate the effect of QRs on shift functioning, and the potential moderating effect of light and specific genotypes.** Finally, the study has ambitions to identify a low threshold offer to shift workers suffering from SWD. In a previous study, we found that Norwegian nurses have little knowledge about the negative consequences associated with shift work.[45] A self-help book for shift workers, written in Norwegian, will therefore be used to examine if knowledge and advice that address specific shift work problems may reduce sickness absence and improve health in healthcare workers with SWD.

1.3 Research questions and hypotheses, theoretical approach and methodology

1.3.1 Work packages (WPs)

WP1: A national payroll register for healthcare workers in the public sector

The main aim in WP1 is to raise the methodological standard in shift work research by establishing a national payroll (shift work) registry and being among the first to examine the effect of seasonal variations in light on the relationship between shift work and sickness absence using an ecological and large national representative sample. *The following research questions (Q) will be addressed:* Q1: What are the prospective effects of night work, evening work, early morning work, long working hours (≥8/10/12 hours), QRs, overtime, rotating 2-shifts, rotating 3-shifts and fixed shifts (evening, night) on healthcare workers' sickness absence? Q2: What are the within-subject effects of exposure to different work schedules (night work, evening work, early morning work, long working hours, QRs, overtime, rotating 2-shifts, rotating 3-shifts, working fixed shifts and change in work schedule) on healthcare workers' sickness absence rates? Q3: What is the predictive effect of age, sex, education level and occupation on shift related sickness absence? Q4: Are there seasonal differences in shift related sickness absence, and do they differ between healthcare workers employed in the northern and southern parts of Norway, and does this vary by season? Q5: Are there differences in shift-related sickness absence in Denmark, Finland and Norway, and are any potential differences related to sex and age?

Approach and choice of method in WP1: In this WP we aim to use the objective payroll and sickness absence records kept by the healthcare workers' employer and investigate the relationship between exposure to different shift characteristics and later sickness absence. The health enterprises store the payroll and sickness absence records retrospectively for at least 5 years, which allows us to perform longitudinal analyses on highly rigorous data. In particular, the records kept by the hospitals include actual dates and start and stop times for every shift the health care workers have carried out, as well as the date of every absence due to sickness they have had over the same 5-year period. Such comprehensive and detailed data presents a vast array of opportunities for innovative and coveted analyses. All four regional health authorities (Central Norway Regional Authority (St Olav hospital), Northern Norway Regional Health Authority (all hospitals), Southern and Eastern Norway Regional Health Authority (all hospitals), Western Norway Regional Health Authority (Stavanger and Haukeland University Hospital) will be invited to contribute data to the national

registry (N=approximately 110 000). Variables to be included are age, sex, occupation, education level (nurse assistant, nurse with BA degree, specialised nurse etc), unit level (medical, surgery, psychiatry), daily work schedules and sickness absence over the last five years. All registry data will be analyzed using methods for repeated observations (e.g. Poison or negative binomial regression models).

WP2: Determining the effect of quick returns in a randomised controlled trial (RCT)

The main aim of WP2 is to determine the effect of QRs in an RCT. The main outcome will be sickness absence, sleep, health and work-related accidents. A secondary aim of WP2 is to examine the role of individual characteristics in the tolerance to QRs. *The following hypotheses (H) will be addressed:* H1: Healthcare workers who are assigned to a work schedule that does not include QRs will have fewer sickness absence days and periods, compared to those who continue in a work schedule that includes QRs. H2: Healthcare workers with schedule that does not include QRs will experience better sleep, physical and psychological health, lower risk of work-related accidents, and reduced turnover intention compared to those with schedule that includes QRs. H3: Individual characteristics associated with shift work tolerance, including sex, age, personality and subjectively reported sleep need, will moderate the negative effects of QRs on the outcomes in H1 and H2. H4: Satisfaction with work schedule, job satisfaction, job engagement and work-family interference will moderate the negative effects of QRs on the outcomes in H1 and H2.

Approach and choice of method in WP2. In collaboration with Haukeland University hospital, we will conduct an RCT to investigate the hypothesis that work schedules that eliminate QRs lead to less sickness absence and turnover, better sleep, health and less work-related accidents. The design and procedure will follow the CONSORT statement and a detailed study protocol will be developed and registered in ClinicalTrials.gov. Healthcare workers who consent to participate will be randomized to either follow a schedule eliminating QRs for 6 months, or to a control group where no changes will be made to their shift schedule. Before the analyses, information from the payroll data will be used to assure compliance with the study protocol. Inclusion criteria will be working 80% of a full time equivalent as a healthcare worker and having a work schedule that includes at least four QRs per month. The necessary sample size (assuming a power of .80 and significance level of .05) has been calculated to be 448 participants in each condition. This calculation is based on the mean values of sickness absence days per month (0.9 days, SD=1.6) as reported in Vedaa et al.[16] The main outcome variable in WP2 will be sickness absence days and spells, which will be retrieved from the local records kept by Haukeland University Hospital. We will compare the sickness absence rates prior to work schedule change, to the abscence during and after the intervention period. Secondary outcome will be self-reported work-related accidents/near accidents, sleep, health complaints, symptoms of anxiety and depression, turnover intention, satisfaction with job and work schedule and work-family interference. Participants will complete questionnaires on demographics and personality, which will be used in moderator analyses. A subsample (n=100) will be invited to use an Data will be analyzed using generalized linear mixed models (for count variables such as sickness absence we will use Poison or negative binomial models). In all analyses, adjustment for individual shift work experience (i.e. number of years working shifts) and history of sickness absence one year prior to the intervention period will be included.

WP3: Adaptation to quick returns The main aim in WP3 is to investigate the effects of QR on objectively recorded sleep and shift function, and to investigate if specific genotypes and personality traits are related to tolerance to QRs. Another aim is to investigate whether a light intervention may counteract the negative effects of QR. *The following hypotheses will be addressed:* H1: Sleep following QR will be curtailed compared to sleep between two-day shifts. H2: Daytime functioning and sleep following QR will be impaired compared to sleep and functioning on a day shift following a preceding day shift. H3: Specific genotypes (rs25531, rs8192440 and rs12506228) and personality traits like morningness[46], flexibility and languidity will moderate the effect of QR on functioning and sleep. H4: A bright light intervention (1000 lux, 4000K will counteract performance decrements following a QR compared to standard ambient illumination (200 lux, 4000 K).

Approach and choice of method in WP3: In a shift work simulation crossover experimental study, subjects will work Day-Day (DD) and Evening-Day (ED). Dayshift starts at 0700 and ends at 1500, whereas evening shift starts at 1500 and ends at 2200. Subjects (nurse students) will be randomized to the order (DD-ED vs. ED-DD). The two consecutive shifts will be spaced by four weeks. Sleep the 3 days before, during and 3 days following the shifts will be assessed by a sleep diary[47] and the Xethru sensor.[48] Each hour during the simulated shifts, the subjects will complete cognitive tests; the Psychomotor Vigilance Test (PVT)[49] and the symbol digit substitution task (SDST)[50]. Subjective measures of affect (PANAS)[51] and sleepiness (Karoliniska Sleepiness Scale)[52] will also be administered hourly. Genotyping will be conducted by custom TaqMan SNP genotyping assays. In a similar experiment using a crossover, design subjects will receive bright light (1000 lux; 4000K) and standard light (200 lux; 4000K) on the dayshift following QR using the

same outcome and moderators as for the first experiment. Data will be analyzed with linear mixed models. Power analyses show that 50 subjects are needed for each experiment when setting the effect size (d) to .40, power to .80, and the correlation between measures to .50.[53]

WP 4: Determining the effect of a self-help book for shift workers in a randomised controlled trial
The main aim in WP4 is to evaluate if a self-help book for coping with shift work[54] can reduce the prevalence of SWD sickness absence, turnover intention, and improve health in healthcare workers. *The following hypotheses will be addressed:* H1: Reading the self-help book will reduce sickness absence compared to those who will receive a paper with general sleep hygiene advices (control condition). H2*:* The self-help book will reduce the prevalence of SWD, improve sleep, health and increased their knowledge about how to prevent negative health consequences associated with shift work compared those who will receive a paper with general sleep hygiene advices (control condition).

Approach and choice of method in WP4: The book concerns three parts; information about sleep and diurnal rhythms, potential consequences of irregular working time and specific advice on how to cope with irregular working time. The book is based on evidence available in the scientific literature up to 2018. Participants will be recruited through the SUSSH study – were nurses with shift work disorders already have been identified. We will conduct an RCT randomizing participants to the self-help book or a paper with general sleep hygiene advices. The design and procedure of the RCT will follow the same procedures as in WP2. Nurses agreeing to participate will be provided with a copy of the self-help book and asked to read it and follow the activities that suit their work schedule and problems. Both groups will be asked to complete a questionnaire at baseline, and at 6 months follow-up. Participants in the control condition will receive the book after the 6 months follow-up period. Inclusion criteria will be holding at least an 80% full time equivalent position as a nurse and fulfilled the diagnostic criteria for SWD. The main outcome variable will be prevalence of SWD, health complaints and subjectively reported sickness absence. Secondary outcome will be satisfaction with the book and knowledge about how to prevent negative consequences of shift work. Data will be analyzed with generalized linear mixed models (Poison or negative binomial models for count variables).

WP5: Implementation of shift work knowledge
The main aim in WP5 is to bridge the gap between research and practice. *The following research questions will be addressed:* Q1: what is best practice to secure that novel and important research about shift work, health and work-related accidents will be communicated to practice and politicians? Q2: How can we use technology and innovative methods to implement findings from research? Q3: How can the health enterprises stimulate researcher to focus on the factors they experience as challenging?
Approach and choice of method in WP5: The Haukeland University Hospital will be responsible for arranging a conference were relevant stakeholders (nurses responsible for developing work schedules, trade unions, politicians and innovators) will be invited to discuss the latest research findings and how implementing of these results should be incorporated into politics and practice.

1.3.2 Instruments: In addition to demography and relevant backgrounds variables, the following validated instruments will be used in WP2, WP3, and WP4: Bergen Insomnia Scale (BIS) will be used to measure insomnia.[55] The Swedish occupational fatigue inventory will be used to measure lack of energy, physical exertion, physical discomfort, lack of motivation and sleepiness.[56] The short version of the Horne-Östberg Morningness-Eveningness Questionnaire will be used to measure diurnal preference.[57] The revised Circadian Type Inventory will be used to measure flexibility and languidity.[58] The Hopkins Symptom Checklist will be used to measure anxiety and depression[59] Shift work disorder will be measured with three standardised questions.[60] Job satisfaction will be measured with the 5 items measuring satisfaction with work.[61] The Work family Interface Scale will be used for detecting work to family balance in either directions.[62] Work-related negative incidents will be assessed using eight items measuring the number of self-reported work-related accidents, near accidents and dozing off at work[63] Turnover intention will be measured with the Turnover Intention Scale as well as three single turnover intention items. In WP2, a subsample will be invited to use assess sleep 4 weeks at baseline and 4 weeks after abolishing QRs with an Actigraphy [64].In WP3, cognitive performance will be measured with the Psychomotor Vigilance Test (PVT)[49] and the symbol digit substitution task (SDST),[50] and the Xethru sensor*,* a low-powered ultra-wideband radar and a sleep diary[65] will be used to assess sleep 3 days before, during and 3 days following the shifts.[48] Affect will be measured with PANAS and the Karolinska Sleepiness Scale will be used to measure sleepiness. In WP4, knowledge

about how to prevent negative health consequences associated with shift work will be measured with 24 multiple choice questions each with four answer options.[45]

***1.3.3 Possible risks**: WP1* is planned as collaboration with all the regional healthcare trusts in Norway. In our pilot study, where we used payroll and sickness absence records kept in the employers' registers, we collaborated with the HR department at Haukeland University hospital and they agree to continue this collaboration. If we receive permission, they will give us access to the data needed, however, we need to establish similar agreements with the other regional health authorities in Norway. The General Data Protection Regulation (GDPR) that applies from May 2018, may cause trouble when the health enterprises are to link payroll and sickness absence data. If this should be the case, the project team will: 1) Ask for permission to obtain data on shift exposure only, without obtaining consent from the participants and rather link the data to sickness absence data from the Norwegian labour and welfare administration. 2) Ask for permission to use payroll data with use of passive consent. The employer informs the employees by email and by pay check. The employees who do not want to participate can contact the employer. *WP2, WP3 and P4*: Possible risks entail recruitment problems, lower than expected effects and equipment malfunction. Risks can be managed with an active and broad recruitment approach as well as compensating participants, by running interim analyses and up-scaling the number of participants if necessary, and thorough equipment checks and establishing equipment backup.
1.3.4 Interdisciplinary: The project team is interdisciplinary and consists of professions including psychologists, a medical doctor, statisticians, a physiologist and nurses with master's degrees in health promoting work.
***1.3.5 Ethical issues**:* The project will be performed in accordance with the Helsinki Declaration and requires permission from the Regional Committee for Medical and Health Research Ethics, Norway as well as permission from the Norwegian Data Protection Authority.
***1.3.6 Gender issues**:* Both genders are included in the sample. However, most healthcare workers are women, and in Norway about 35% of women and 30% of men work shifts. Therefore, the project may be somewhat more relevant for women than men overall.
***1.3.7 Environmental impact**:* The findings from the project may potentially pave the way for positive changes in terms of working conditions, especially pertaining to shift work. Thus, the project and its results will be beneficial for the working environment.
1.3.8. Data handling: Research data in HeWoS will be stored at UiB on a new platform called SAFE (Secure Access to Research Data and E-infrastructure). Scientific publications will be published in open access journals or available through the institutional (UiB) online research archive (BORA).

2. Impact

2.1. Societal, practical and scientific impact

Societal: Lack of qualified health personnel is estimated to be a significant society challenge in the future. Thus, HeWoS has implications for **UN Sustainable development goal (SDG#3**), Ensure healthy lives and promote well-being for all at all ages. If successful, the project outputs will give a significant contribution to a sustainable work force in the health and care sector. HeWoS will therefore also address **SDG#8** Promote sustained, inclusive and sustainable economic growth, full and productive employment and decent work for all. Outputs from the register study will be especially useful for policy makers and those planning work schedules as it offers detailed information about how all the different shift pattern and the distribution of rest period is associated to sickness absence. Based on outputs from the interventions investigating effects of QRs (WP2 and WP3) relevant stakeholders will get evidence to support their solutions. They will get knowledge if there are groups of workers who are more vulnerable or resistance to QR. The interventions will indicate if investment in more advanced light system is a good idea. If effective, outputs from WP4 can be used to help workers with SWD. The sleep advices and the book are already available. However, if effective, the employers should consider spreading this knowledge to help up to 44% of the nursing population.
***Scientific*:** HeWoS will investigate the link between shift work and impaired health, using reliable and accurate assessments of exposure and outcomes in a register study and RCTs. In a future perspective, the register data can be updated and linked to other national health registers. This is a unique opportunity to study long-term consequences due to shiftwork. BThe genetic differences in tolerance to QRs and the natural differences in daylight exposure offer a long-term population study that could contribute to a deeper understanding of underlying mechanism in the development of negative health outcomes.

2.3 Measures for communication and exploitation

The output from HeWoS is especially relevant for shift workers in the healthcare sector, but also for all employees, employers, trade unions, and policy makers who are engaged in shift work. In order to secure implementation of results the project group will work in close collaboration with the large health enterprises in Norway. The HR department in the Haukeland University Hospital will arrange a conference where relevant stakeholders will be invited (WP5). On the conference, relevant outputs and implementation of results in practice and politics will be discussed through panel sessions and workshops. In addition, all health enterprises that brings data into the register base will receive a report summarizing the main results. The report will be written by the researchers in collaboration with representatives from the user group and point to countermeasures aiming to prevent negative health outcomes and accidents. A webpage for HeWoS will be set up at www.BeSCN.no. Dissemination of results linked to national and international publications will be available at this webpage. The findings will be published in popular science publications like "Søvn" and "Sykepleien", which are relevant journals for the target audience. A user group comprising members from the workers unions and employer organization within the healthcare enterprises will be established. Annual meetings between the research team and the user group will be arranged. PhD and postdoc candidates are expected to publish three and five papers respectively. The papers will address the objectives listed in this proposal. The findings will be reported in high-impact peer reviewed international journal.

3 Implementation

3.1 Project management and project group

Anette Harris, principal investigator (PI) of this grant proposal, is a Professor in work and organizational psychology. She has worked on several RCTs aiming to reduce or prevent sickness absence and was PI in the pilot study using payroll data to explore associations between shift work and sickness absence. Since she defended her thesis in 2011, she has/is supervising 7 PhD-candidates (4 defended). Harris is a head of the interdisciplinary research group Bergen Sleep and Chronobiology network (www.BeSCN.no).
Ståle Pallesen, Professor in Psychology (PhD) is an accredited somnologist and has been PI for several sleep related projects, included light and lab-based interventions. He has received funding for several projects from the Norwegian Research Council, and has/is supervising about 50 PhD-candidates and has a high scientific output (no 33 in Norway in 2017;www.khrono.no/files/2017/11/15/topp100-siste-5-ar.pdf; H-index ResearchGate: 52).
Bjørn Bjorvatn, Professor in Medicine, is a certified sleep expert and has led several large sleep related projects. Bjorvatn is a director of the Norwegian Competence Center for Sleep Disorders. He has written 5 textbooks in Norwegian (one a revision), 38 chapters in Norwegian textbooks, 222 publications in Norwegian and 198 original peer-reviewed articles in English (1989-2018), 12 review papers and 10 chapters in English textbooks (among them two chapters in the official European textbook on sleep). H-index ResearchGate: 51. Bjorvatn is the author of the self-help book on how to cope with shift work, which is tested in WP4.
Øystein Vedaa is a director of the Department of Health Promotion at the Norwegian Institute of Public Health and a researcher at the Department of Mental Health, Norwegian University of Science and Technology (NTNU). Vedaa is currently a PI of an ongoing field study on shift work and the effects of blue-depleted indoor lighting during evening and night shifts for hospital nurses. He has experience in handling shift work registry data from the Norwegian health enterprises and has used such data in several publications. Vedaa has experience in conducting randomized controlled treatment trials for sleep disorders.
Siri Waage, is a Post doctor at UiB and the coordinator of the Norwegian Competence Centre for Sleep Disorders. For the last six years, Waage has acted as the coordinator of the Survey of Shift Work, sleep and Health (SUSSH) study, a longitudinal cohort among nearly 2900 Norwegian nurses. Waage has a PhD within the research field of shift work and published 38 international scientific papers within this field. Waage is currently supervising and co-supervising 3 PhD candidates within shift work research.
Stein Atle Lie, Professor at the University of Bergen. Lie is a statistician and has worked with various projects analysing sickness absence and work in Norway and other Scandinavian countries and introduced multi-state models for that purpose. He has written 141 scientific articles in international per-rewieved journals. H-index: Web of Science: 39.
Morten Birkeland Nielsen, Professor in psychology and research director at the National institute of occupational Health (STAM). Nielsen is a national partner who has worked with various epidemiological projects within this field. He has written 90 scientific articles (43 as first author) since 2005. H-index: 34, i10-index:48.

Erling Svensen, Cand Psychol, PhD, is a senior adviser at the HR department at Haukeland University Hospital. He is an experience work and organizational psychologist working within areas such as change management and organizational development. The research team has collaborated with Svensen since 2008.
Anne Helene Garde, Research Professor, is an international partner from the National Research Centre for the Working Environment in Denmark. Garde is a specialist within psychosocial work environments and health. Her work is mainly focussed around working hours, sleep and stress. She is currently head of the network "Working hours In the Nordic Countries" (WINC) and her scientific production includes 115 peer reviewed scientific papers in the field of physiology, chronobiology and epidemiology. The research team has collaborated with Garde since 2008.
Annina Ropponen, Senior researcher and adjunct professor is an international partner from the Finnish Institute of Occupational Health (FIOH). Ropponen has long experience of longitudinal, epidemiological studies of sickness absence and disability pensions and has been supervisor of 3 PhDs within the field. She has the core role in the development and analyses of Finnish pay-roll based working hour database. The research team has collaborated with Ropponen since 2015.

3.2 Project organization and management

Organization and management structure:

The project will be anchored at the Department of Psychosocial Science, Faculty of Psychology, UiB. Administration of the grants follows routines of the Faculty of Psychology, UiB. The three candidates and their supervisors will form a close team, with regular joint research meetings in the interdisciplinary research group BeSCN (www.BeSCN.no) and the Norwegian Competence Center for Sleep Disorders, Bergen. Meetings with national and international partners will be arranged annually. International collaboration with other shift work researchers have been formalised since 2008, as part of the SUSSH study, managed by Pallesen, Bjorvatn, and Waage, and from 2013-2014 in the WINC network, managed by Garde and currently in the a Scandinavian network funded by NordForsk: Working hours, health, well-being and participation in working life (WOW), managed by Professor Härmä at FIOH. The research group has collaboration with Haukeland University hospital, especially the Human Resource Department since 2014 and with senior adviser Erling Svensen since 2008.

The establishing of a national shift work register based on payroll data will be organized from UiB by the post.doc under supervision of Harris. The statistical analyses of payroll data regarding comparison of shift related sickness absence in Denmark, Finland and Norway will be performed by the postdoc candidate under supervision of Garde, Ropponen, Harris, Vedaa and Lee. The reference group in all three countries will participate in the discussion and writing of the paper. The postdoc candidate will spend at least two months in Denmark and two months in Finland under supervision of Garde and Ropponen.
The RCTs investigating the effect of a schedule without QRs and the effect of a self-help book for shift workers suffering from SWD will be organized from UiB and led by the first PhD candidate under supervision of Vedaa. The shift work simulation study investigating the effect of QRs on shift functioning, and the potential moderating effect of light and specific genotypes will be organized by UiB and led by the second PhD candidate under supervision of Pallesen. All partners will participate in supervising and co-authoring papers. WP5 will be organized by the health enterprises, Haukeland University Hospital and led by the senior adviser Svensen. All members in the research group will contribute to the conference.
Infrastructure: Most of the necessary infrastructure is in place. The research group has available actiwatches, however due to other on-going studies, a supplement of 10 actigraphs are needed. The faculty of Psychology has already established a lab where we can simulate QRs under different light conditions. The Xethru sensor will be borrowed from the NTNU.
Organization and management structure: The project will be anchored at the Department of Psychosocial Science, Faculty of Psychology, UiB. Administration of the grants follows routines of the Faculty of Psychology, UiB. The three candidates and their supervisors will form a close team, with regular joint research meetings in the interdisciplinary research group BeSCN (www.BeSCN.no) and the Norwegian Competence Centre for Sleep Disorders, Bergen. Meetings with national and international partners will be arranged annually. International collaboration with other shift work researchers have been formalised since 2008, as part of the Survey of Shift Work, sleep and Health (SUSSH), managed by Pallesen, Bjorvatn, and Waage, and from 2013-2014 in the Nordic network: Working hours In the Nordic Countries (WINC),

managed by Garde and currently in the Scandinavian network funded by NordForsk: Working hours, health, well-being and participation in working life (WOW), managed by Professor Härmä in Finland.

References:

1.Parent-Thirion A, Fernández Macías, E, Hurley J, Vermeylen G. *Fourth European workingconditions survey.* 2007.
2.StatisticsNorway. Sickness absence. http://www.ssb.no/en Accessed 06.04.2018, 2018.
3.Kovner CT, Brewer CS, Fatehi F, Jun J. What Does Nurse Turnover Rate Mean and What Is the Rate? Policy, Politics, & Nursing Practice. 2014; 15: 64–71.
4.Norway S. Nurses' workplace and newly graduated nurses affiliation with working life. https://www.ssb.no/en/helse/artikler-og-publikasjoner/nurses-workplace-and-newly-graduated-nurses-affiliation-with-working-life. Accessed 06.04.19, 2019.
5.Flinkman M, Laine M, Leino-Kilpi H, Hasselhorn HM, Salantera S. Explaining young registered Finnish nurses' intention to leave the profession: a questionnaire survey. Int J Nurs Stud. 2008; 45 (5): 727-739.
6.Czeisler CA, Duffy JF, Shanahan TL, et al. Stability, precision, and near-24-hour period of the human circadian pacemaker. Science. 1999; 284: 2177-2181.
7.Johnson CH, Elliott JA, Foster R. Entrainment of circadian programs. Chronobiol Int. 2003; 20: 741-774.
8.Khalsa SBS, Jewett ME, Cajochen C, Czeisler CA. A phase response curve to single bright light pulses in human subjects. J Physiol. 2003; 549: 945-952.
9.Kecklund G, Axelsson J. Health consequences of shift work and insufficient sleep. Bmj-Brit Med J. 2016; 355.
10.Sateia MJ. International Classification of Sleep Disorders-Third Edition Highlights and Modifications. Chest. 2014; 146 (5): 1387-1394.
11.Asaoka S, Aritake S, Komada Y, et al. Factors associated with shift work disorder in nurses working with rapid-rotation schedules in Japan: the nurses' sleep health project. Chronobiology international. 2013; 30 (4): 628-636.
12.Flo E, Pallesen S, Mageroy N, et al. Shift Work Disorder in Nurses - Assessment, Prevalence and Related Health Problems. Plos One. 2012; 7 (4).
13.Bjorvatn B, Pallesen S, Moen BE, Waage S, Kristoffersen ES. Migraine, tension-type headache and medication-overuse headache in a large population of shift working nurses: a cross-sectional study in Norway. Bmj Open. 2018; 8 (11): e022403.
14.Waage S, Pallesen S, Moen BE, Bjorvatn B. Restless Legs Syndrome/Willis-Ekbom Disease Is Prevalent in Working Nurses, but Seems Not to Be Associated with Shift Work Schedules. Front Neurol. 2018; 9: 21.
15.Niedhammer I, Lert F, Marne MJ. Effects of shift work on sleep among French nurses - a longitudinal study. J Occup Environ Med. 1994; 36: 667-674.
16.Vedaa O, Pallesen S, Waage S, et al. Short rest between shift intervals increases the risk of sick leave: a prospective registry study. Occup Environ Med. 2017; 74 (7): 496-501.
17.Eldevik MF, Flo E, Moen BE, Pallesen S, Bjorvatn B. Insomnia, Excessive Sleepiness, Excessive Fatigue, Anxiety, Depression and Shift Work Disorder in Nurses Having Less than 11 Hours in-Between Shifts. PLoS One. 2013; 8: e70882.
18.Garde AH, Harris A, Vedaa Ø, et al. Working hour characteristics and schedules among nurses in three Nordic countries - a comparative study using payroll data. BMC Nursing. 2019; (18:12).
19.Åkerstedt T, Ingre M, Kecklund G. *Vad kännetecknar bra och dåliga skiftscheman? Stressforskningsrapporter nr 324.* Stressforskningsinstitutet, Stockholms universitet;2012.
20.Dahlgren A, Tucker P, Gustavsson P, Rudman A. Quick returns and night work as predictors of sleep quality, fatigue, work–family balance and satisfaction with work hours. Chronobiology international. 2016: 1-9.
21.Dahlgren A, Tucker P, Gustavsson P, Rudman A. Quick returns and night work as predictors of sleep quality, fatigue, work-family balance and satisfaction with work hours. Chronobiology international. 2016; 33 (6): 759-767.
22.Vedaa O, Morland E, Larsen M, et al. Sleep Detriments Associated With Quick Returns in Rotating Shift Work: A Diary Study. J Occup Environ Med. 2017; 59 (6): 522-527.
23.Flo E, Pallesen S, Moen BE, Waage S, Bjorvatn B. Short rest periods between work shifts predict sleep and health problems in nurses at 1-year follow-up. Occup Environ Med. 2014; 71 (8): 555-561.
24.Nielsen HB, Hansen ÅM, Conway SH, et al. Short time between shifts and risk of injury among Danish hospital workers: a register-based cohort study. Scandinavian Journal of Work, Environment & Health. 2018.
25.Hensing G, Alexanderson K, Allebeck P, Bjurulf P. How to measure sickness absence? Literature review and suggestion of five basic measures. Scand J Soc Med. 1998; 26 (2): 133-144.
26.Vester TS, Claus F, Birgitte L, et al. *Sickness Absence in the Nordic Countries.* 2015.
27.Ropponen A, Koskinen A, Puttonen S, Harma M. Exposure to working-hour characteristics and short sickness absence in hospital workers: A case-crossover study using objective data. Int J Nurs Stud. 2019; 91: 14-21.
28.Merkus SL, van Drongelen A, Holte KA, et al. The association between shift work and sick leave: a systematic review. Occup Environ Med. 2012; 69 (10): 701-712.
29.van Drongelen A, Boot CR, Hlobil H, van der Beek AJ, Smid T. Cumulative exposure to shift work and sickness absence: associations in a five-year historic cohort. BMC Public Health. 2017; 17 (1): 67.
30.Vedaa O, Pallesen S, Waage S, et al. Short rest between shift intervals increases the risk of sick leave: a prospective registry study. Occup Environ Med. 2016.
31.Saksvik-Lehouillier I, Bjorvatn B, Hetland H, et al. Individual, situational and lifestyle factors related to shift work tolerance among nurses who are new to and experienced in night work. J Adv Nurs. 2013; 69 (5): 1136-1146.
32.Saksvik IB, Bjorvatn B, Hetland H, Sandal GM, Pallesen S. Individual differences in tolerance to shift work - a systematic review. Sleep medicine reviews. 2011; 15: 221-235.
33.Di Milia L, Smith PA, Folkard S. A validation of the revised circadian type inventory in a working sample. Personality and Individual Differences. 2005; 39: 1293-1305.
34.Reszka E, Peplonska B, Wieczorek E, et al. Rotating night shift work and polymorphism of genes important for the regulation of circadian rhythm. Scandinavian journal of work, environment & health. 2013; 39 (2): 178-186.

35.Thun E, Le Hellard S, Osland TM, et al. Circadian clock gene variants and insomnia, sleepiness, and shift work disorder. Sleep and Biological Rhythms. 2016; 14 (1): 55-62.
36.Sulkava S, Ollila HM, Alasaari J, et al. Common Genetic Variation Near Melatonin Receptor 1A Gene Linked to Job-Related Exhaustion in Shift Workers. Sleep. 2017; 40 (1).
37.Pallesen S, Jacobsen DP, Nielsen MB, Gjerstad J. The genpotype 5HTTLPR rs25531 LALA is associated with insomnia in shift work. Sleep 2019 (resubmitted).
38.Folkard S, Tucker P. Shift work, safety and productivity. Occup Med. 2003; 53: 95-101.
39.Wagstaff AS, Lie JAS. Shift and night work and long working hours - a systematic review of safety implications. Scandinavian Journal of Work Environment & Health. 2011; 37: 173-185.
40.Roenneberg T, Foster RG. Twilight times: light and the circadian system. Photochem Photobiol. 1997; 66 (5): 549-561.
41.Bjorvatn B, Kecklund G, Akerstedt T. Rapid adaptation to night work at an oil platform, but slow readaptation after returning home. J Occup Environ Med. 1998; 40 (7): 601-608.
42.Harris A, Waage S, Holger U, Hansen AM, Bjorvatn B, Eriksen HR. Shift Work, Cortisol, Reaction Time Test and Health among Offshore Workers. International Journal of Behavioral Medicine. 2010; 17: 88-88.
43.Lie SA, Tveito TH, Reme SE, Eriksen HR. IQ and mental health are vital predictors of work drop out and early mortality. Multi-state analyses of Norwegian male conscripts. PLoS One. 2017; 12 (7): e0180737.
44.Gran JM, Lie SA, Oyeflaten I, Borgan O, Aalen OO. Causal inference in multi-state models-sickness absence and work for 1145 participants after work rehabilitation. BMC Public Health. 2015; 15: 1082.
45.Warpe H, Bjorvatn B, Waage S, Hovlid E, Pallesen S. Vet lite om helsefarene ved turnusarbeid. Sykepleien. 2013; 101 (11): 54-57.
46.Horne JA, Ostberg O. A self-assessment questionnaire to determine morningness-eveningness in human circadian rhythms. International journal of chronobiology. 1976; 4 (2): 97-110.
47.Lichstein KL, Riedel BW. Behavioral-Assessment and Treatment of Insomnia - a Review with an Emphasis on Clinical-Application. Behavior Therapy. 1994; 25 (4): 659-688.
48.Pallesen S, Gronli J, Myhre K, et al. A Pilot Study of Impulse Radio Ultra Wideband Radar Technology as a New Tool for Sleep Assessment. Journal of Clinical Sleep Medicine. 2018; 14 (7): 1249-1254.
49.Basner M, Dinges DF. Maximizing Sensitivity of the Psychomotor Vigilance Test (PVT) to Sleep Loss. Sleep. 2011; 34 (5): 581-591.
50.Royer FL, Gilmore GC, Gruhn JJ. Normative Data for the Symbol Digit Substitution Task. Journal of clinical psychology. 1981; 37 (3): 608-614.
51.Watson D, Clark LA, Tellegen A. Development and validation of brief measures of positive and negative affect: the PANAS scales. J Pers Soc Psychol. 1988; 54 (6): 1063-1070.
52.Kaida K, Takahashi M, Akerstedt T, et al. Validation of the Karolinska sleepiness scale against performance and EEG variables. Clin Neurophysiol. 2006; 117 (7): 1574-1581.
53.Faul F, Erdfelder E, Lang AG, Buchner A. G*Power 3: a flexible statistical power analysis program for the social, behavioral, and biomedical sciences. Behavior research methods. 2007; 39 (2): 175-191.
54.Bjorvatn B. *Skiftarbeid og søvn. Slik mestrer du nattarbeid og uregelmessig arbeidstid.* Fagbokforlaget; 2019.
55.Pallesen S, Bjorvatn B, Nordhus IH, Sivertsen B, Hjornevik M, Morin CM. A new scale for measuring insomnia: the Bergen Insomnia Scale. Percept Mot Skills. 2008; 107 (3): 691-706.
56.Ahsberg E, Gamberale F, Gustafsson K. Perceived fatigue after mental work: an experimental evaluation of a fatigue inventory. Ergonomics. 2000; 43 (2): 252-268.
57.Adan A, Almirall H. Horne and Ostberg Morningness Eveningness Questionnaire - a Reduced Scale. Pers Indiv Differ. 1991; 12 (3): 241-253.
58.Di Milia L, Smith PA, Folkard S. A validation of the revised circadian type inventory in a working sample. Pers Indiv Differ. 2005; 39 (7): 1293-1305.
59.Derogatis LR, Lipman RS, Rickels K, Uhlenhuth EH, Covi L. The Hopkins Symptom Checklist (HSCL). A measure of primary symptom dimensions. Mod Probl Pharmacopsychiatry. 1974; 7 (0): 79-110.
60.Waage S, Moen BE, Pallesen S, et al. Shift work disorder among oil rig workers in the North Sea. Sleep. 2009; 32 (4): 558-565.
61.Quinn RP, Shepard LJ. Job satisfaction. Ann Arbor, MI, US: University of Michigan, Institute for Social Research, 1974 328 pp. 1974.
62.Curbow B, McDonnell K, Spratt K, Griffin J, Agnew J. Development of the Work-Family Interface Scale. Early Child Res Q. 2003; 18 (3): 310-330.
63.Vedaa O, Harris A, Erevik EK, et al. Short rest between shifts (quick returns) and night work is associated with work-related accidents. Int Arch Occup Environ Health. 2019.
64.Sadeh A. The role and validity of actigraphy in sleep medicine: an update. Sleep Med Rev. 2011; 15 (4): 259-267.
65.Lichstein KL, Wilson NM, Noe SL, Aguillard RN, Bellur SN. Daytime sleepiness in insomnia: behavioral, biological and subjective indices. Sleep. 1994; 17 (8): 693-702.
