# Supplementary material for: Shift Schedule With Fewer Short Daily Rest Periods and Sickness Absence Among Health Care Workers: A Cluster Randomized Clinical Trial
Source: JAMA Netw Open. 2025 Sep 15;8(9):e2531568. doi: 10.1001/jamanetworkopen.2025.31568 (PMC12439055; doi:10.1001/jamanetworkopen.2025.31568)
Supplement: Supplement 2. — eFigure. Observed Mean Sickness Absence Days per Month During the 6-Month Reference and Intervention Periods, Stratified by Randomization Group for Healthcare Workers Working Equivalent to ≥ 80% of a Full-Time Position eTable 1. Table Outlining the Changes to the Study During the Project Period Due to the COVID-19 Pandemic eTable 2. Examples of a Two-Week Cycle of Rotating Shift Work With and Without Short Daily Rest Periods (<11 Hours off Between Two Consecutive Shifts) eAppendix 1. Sensitivity Analyses eTable 3. Results From the Sensitivity Analyses Where the Intervention Effect Was Adjusted for the Baseline (i.e., Reference Period) Value of Sickness Absence and Analyses Also Using Flat Negative Binomial Models With Robust Standard Errors and Baseline Adjustment in Healthcare Workers Who Worked Equivalent to ≥80% of a Full-Time Position (N = 811) eAppendix 2. ITT and Sensitivity Analyses in the ≥50% Position Sample eTable 4. Results From Intention-to-Treat and Sensitivity Analyses on Primary Outcomes on Healthcare Workers Who Worked Equivalent to ≥50% of a Full-Time Position (n=1,764) eAppendix 3. Supplementary Cost-Benefit Analysis eTable 5. Elements in the Calculation of the Net Present Value (NPV) of Economic Returns for the Society From a Shift Schedule With a Reduced Number of Short Daily Rest Periods for Healthcare Workers Working Equivalent to ≥50% of a Full-Time Position eTable 6. Total Number of Responses and Frequency Distribution to Questions About Possible Unwanted Negative Events or Effects as a Result of the Shift Schedule Over the Last Few Months. Responses Were Given at the End of the Six-Month Intervention Period (n=600) eTable 7. Cluster-Adjusted Ordinal-Logistic Regression Results for Self-Reported Questions About Possible Unwanted Negative Events or Effects as a Result of the Shift Schedule the Last Few Months at Six-Month Follow-up (Intervention vs Control) [file jamanetwopen-e2531568-s002.pdf]

Supplementary Online Content

Djupedal ILR, Harris A, Svensen E, et al. Effects of a shift schedule with fewer short daily rest periods on sickness absence among health care workers: a cluster randomized clinical trial. *JAMA Netw Open.* 2025;8(9):e2531568. doi:10.1001/jamanetworkopen.2025.31568

eFigure. Observed Mean Sickness Absence Days per Month During the 6-Month Reference and Intervention Periods, Stratified by Randomization Group for Healthcare Workers Working Equivalent to ≥ 80% of a Full-Time Position
eTable 1. Table Outlining the Changes to the Study During the Project Period Due to the COVID-19 Pandemic
eTable 2. Examples of a Two-Week Cycle of Rotating Shift Work With and Without Short Daily Rest Periods (<11 Hours off Between Two Consecutive Shifts)
eAppendix 1. Sensitivity Analyses
eTable 3. Results From the Sensitivity Analyses Where the Intervention Effect Was Adjusted for the Baseline (i.e., Reference Period) Value of Sickness Absence and Analyses Also Using Flat Negative Binomial Models With Robust Standard Errors and Baseline Adjustment in Healthcare Workers Who Worked Equivalent to ≥80% of a Full-Time Position (N = 811)
eAppendix 2. ITT and Sensitivity Analyses in the ≥50% Position Sample
eTable 4. Results From Intention-to-Treat and Sensitivity Analyses on Primary Outcomes on Healthcare Workers Who Worked Equivalent to ≥50% of a Full-Time Position (n=1,764)
eAppendix 3. Supplementary Cost-Benefit Analysis
eTable 5. Elements in the Calculation of the Net Present Value (NPV) of Economic Returns for the Society From a Shift Schedule With a Reduced Number of Short Daily Rest Periods for Healthcare Workers Working Equivalent to ≥50% of a Full-Time Position
eTable 6. Total Number of Responses and Frequency Distribution to Questions About Possible Unwanted Negative Events or Effects as a Result of the Shift Schedule Over the Last Few Months. Responses Were Given at the End of the Six-Month Intervention Period (n=600)
eTable 7. Cluster-Adjusted Ordinal-Logistic Regression Results for Self-Reported Questions About Possible Unwanted Negative Events or Effects as a Result of the Shift Schedule the Last Few Months at Six-Month Follow-up (Intervention vs Control)

This supplementary material has been provided by the authors to give readers additional information about their work.

Sickness absence days per month

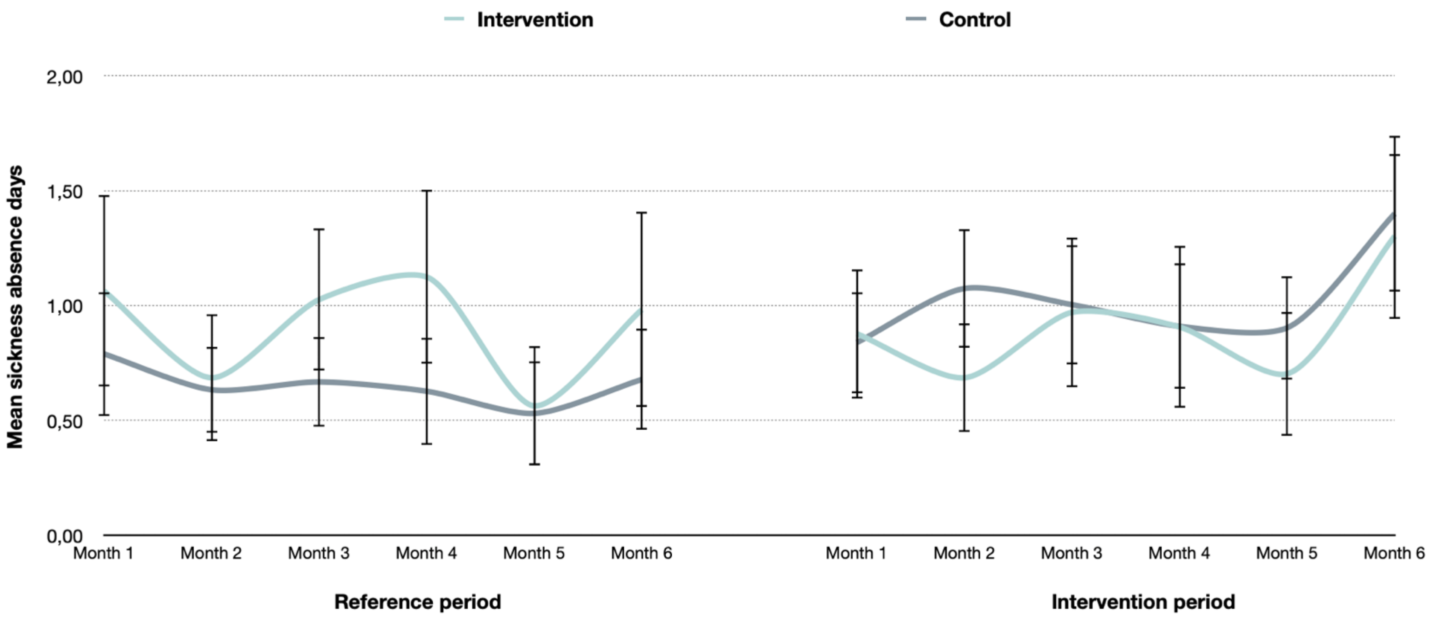

eFigure. Observed Mean Sickness Absence Days per Month During the 6-Month Reference and Intervention Periods, Stratified by Randomization Group for Healthcare Workers Working Equivalent to ≥ 80% of a Full-Time Position

Error bars represent 95% confidence intervals. The reference period reflects the same calendar months one year earlier to account for seasonal variation. Intervention units began implementation at different time points between January 11, 2021, and May 22, 2022; hence, months are labeled generically (Month 1–6), with Month 1 marking the start of the intervention period. Control units were matched to intervention units by size and medical function.

© 2025 Djupedal ILR et al. *JAMA Network Open.*

Changes to the study

eTable 1. Table Outlining the Changes to the Study During the Project Period Due to the COVID-19 Pandemic

Change to the study	Details of the change	Date of change
Change in study population	The intensive care units at Haukeland University hospital were responsible for the treatment of patients with covid-infection during the pandemic. Prior to the randomization, these units gave feedback that it would be difficult to implement and comply to a new shift schedule as the units were in a pressured work situation where considerations of life and health were prioritized. The intensive care units were therefore excluded from the study.	August 2020
Change in sample size	Initially, according to the protocol paper, the intention was to enroll employees who worked ≥80% of a full-time position). However, a lower number than anticipated met this criterion (n=811). In fact, out of the 3,392 individuals who had data for both the reference and intervention periods, the average percentage of full-time positions was just over 60%. To increase the statistical power and ensure that the trial represents the majority of healthcare workers, the decision was made to include all employees who worked ≥50% of a full-time position. The threshold of ≥50% of a full-time position was also pre-registered for the trial on the Clinical Trials website (NCT04693182).	November 2022

Examples of shift schedules with and without short daily rest periods

eTable 2. Examples of a Two-Week Cycle of Rotating Shift Work With and Without Short Daily Rest Periods (<11 Hours off Between Two Consecutive Shifts)

	Week 1							Week 2						
	Monday	Tuesday	Wednesday	Thursday	Friday	Saturday	Sunday	Monday	Tuesday	Wednesday	Thursday	Friday	Saturday	Sunday
Scenario 1: Three-shift rotation[a]														
**with** short daily rest periods	D	D	N	N				E	D	D		E	D	E
**without** short daily rest periods	D	D	N	N				D	D	D		E	E	E
Scenario 2: Three-shift rotation[a]														
**with** short daily rest periods	E	D	D		N	N	N			E	D	D		
**without** short daily rest periods	D	E	E		N	N	N			D	D	D		
Scenario 3: Weekend shift														
**with** short daily rest periods	E	D	D		E	D	E	D		D	D			
**without** short daily rest periods	D	D	D		D	E	E		D	D	E			
Scenario 4: Two-shift rotation[b]														
**with** short daily rest periods		D	D	E	D			E	D	E		D		
**without** short daily rest periods	E		D	D	D			E	E		D	D		

D = Day shift, E = Evening shift, N = Night shift

[a] Three-shift rotation refers to a shift schedule in which the workers alternate between day-, evening- and night shifts.

[b] Two-shift rotation refers to a shift schedule in which the workers alternate between only two of the shifts (e.g., only working day and evening shifts).

© 2025 Djupedal ILR et al. *JAMA Network Open.*

eAppendix 1. Sensitivity Analyses

Post hoc sensitivity analyses for the primary outcomes were conducted, including: (1) Adjustment for the baseline value of the outcome (i.e., number of sickness absence days in the last five months of the reference period). The analysis was performed by including the baseline value as a covariate in the negative binomial mixed-effects model. (2) Fitted negative binomial regression models without random effects (i.e., using a "flat" model), where robust (sandwich) standard errors were estimated, also including baseline adjustment. (3) Adjusting for randomization stratification by incorporating the 10-level randomization stratification factor as a covariate in the mixed effects negative binomial model.

Results from sensitivity analyses adjusting for the baseline value of the outcome (1) using flat negative binomial models with robust standard errors (2) are reported in eTable 3. Results from sensitivity analyses, including the 10-level randomization stratification factor as an additional covariate in the mixed negative-binomial models (3), produced effect estimates comparable to the primary analysis (e.g., sickness absence days: IRR = 0.57, 95% CI = 0.41 to 0.79, $p < 0.001$; sickness absence spells: IRR = 0.73, 95% CI = 0.61 to 0.86, $p < 0.001$).

© 2025 Djupedal ILR et al. *JAMA Network Open.*

eTable 3. Results From the Sensitivity Analyses Where the Intervention Effect Was Adjusted for the Baseline (i.e., Reference Period) Value of Sickness Absence and Analyses Also Using Flat Negative Binomial Models With Robust Standard Errors and Baseline Adjustment in Healthcare Workers Who Worked Equivalent to ≥80% of a Full-Time Position (N = 811)

	**Shift schedule with reduced number of short daily rest periods**		**Shift schedule maintaining the usual number of short daily rest periods**		**(1) Intervention effect adjusted for the baseline value of the outcome**		**(2) Using flat negative binomial models with robust standard errors and baseline adjustment**	
	No.	mean (±SE)[c]	No.	mean (±SE)[c]	IRR (95% CI)	*p*-value	IRR (95% CI)	*p*-value
**Sickness absence days**[a]								
Reference period	334	3.91 (0.56)	467	2.64 (0.35)	**0.68**	**0.013**	**0.74**	**0.049**
Intervention period	334	4.51 (0.64)	467	5.40 (0.68)	(0.51 to 0.92)		(0.55 to 0.99)	
**Sickness absence spells**[b]								
Reference period	334	1.10 (0.10)	467	0.98 (0.08)	**0.74**	**0.001**	**0.74**	**<0.001**
Intervention period	334	1.33 (0.12)	467	1.62 (0.13)	(0.61 to 0.88)		(0.61 to 0.89)	

Mean, predicted marginal mean; SE, Standard error; IRR, Incident rate ratio; CI, confidence interval

[a] Sickness absence during the last 5 months of the reference period and last 5 months of intervention period.

[b] Sickness absence spells (i.e. each uninterrupted period of one or more consecutive sickness-absence days) during the last 5 months of the reference period and last 5 months of intervention period.

[c] Predicted marginal (population-averaged) means from the mixed-effects negative-binomial model; random intercept variances for unit and employee were integrated out before back-transformation.

© 2025 Djupedal ILR et al. *JAMA Network Open.*

eAppendix 2. ITT and Sensitivity Analyses in the ≥50% Position Sample

Post hoc sensitivity analyses for the primary outcomes were conducted, including: (1) Adjustment for the baseline value of the outcome (i.e., number of sickness absence days in the last five months of the reference period). The analysis was performed by including the baseline value as a covariate in the negative binomial mixed-effects model. (2) Fitted negative binomial regression models without random effects (i.e., using a “flat” model), where robust (sandwich) standard errors were estimated, also including baseline adjustment.

© 2025 Djupedal ILR et al. *JAMA Network Open.*

eTable 4. Results From Intention-to-Treat and Sensitivity Analyses on Primary Outcomes on Healthcare Workers Who Worked Equivalent to ≥50% of a Full-Time Position (n=1,764)

	**Shift schedule with reduced number of short daily rest periods**		**Shift schedule maintaining the usual number of short daily rest periods**		**Intervention effect**		**(1) Intervention effect adjusted for the baseline value of the outcome**		**(2) Using flat negative binomial models with robust standard errors and baseline adjustment**	
	No.	mean (±SE)[c]	No.	mean (±SE)[c]	IRR (95% CI)	*p*-value	IRR (95% CI)	*p*-value	IRR (95% CI)	*p*-value
**Sickness absence days**[a]										
Reference period	788	6.76 (0.87)	976	4.85 (0.56)	**0.65** (0.52 to 0.82)	**<0.001**	**0.71** (0.57 to 0.88)	**0.002**	**0.78** (0.63 to 0.96)	**0.033**
Intervention period	788	9.32 (1.19)	976	10.25 (1.21)						
**Sickness absence spells**[b]										
Reference period	788	1.36 (0.11)	976	1.17 (0.09)	**0.82** (0.74 to 0.92)	**<0.001**	**0.82** (0.73 to 0.93)	**0.001**	**0.82** (0.73 to 0.93)	**<0.001**
Intervention period	788	1.73 (0.13)	976	1.82 (0.13)						

Mean, predicted marginal mean; SE, Standard error; IRR, Incident rate ratio; CI, confidence interval

[a] Sickness absence during the last 5 months of the reference period and last 5 months of intervention period.

[b] Sickness absence spells (i.e. each uninterrupted period of one or more consecutive sickness-absence days) during the last 5 months of the reference period and last 5 months of intervention period

[c] Predicted marginal (population-averaged) means from the mixed-effects negative-binomial model; random intercept variances for unit and employee were integrated out before back-transformation.

© 2025 Djupedal ILR et al. *JAMA Network Open.*

eAppendix 3. Supplementary Cost-Benefit Analysis

Description of elements in the cost-benefit analyses: The average annual gross wage for full time position for healthcare workers at the hospital in 2023 amounted to NOK 630,000 (USD ~57,000). The annual value of production (W_{it}) generated by *one* employee (*i)* is represented by the costs for the employer of having the worker in a full-time position throughout a calendar year (t). This is calculated as the sum of the annual wage and other annual employee-specific costs such as payroll tax, social security contributions and potential social costs. If the worker is on sickness absence for the entire year, this will lead to a loss in economic returns for the society amounting to the sum of the annual value of production and the dead weight loss from funding social insurance benefits (in this case sickness benefits) by taxation ($W_{it} + \lambda TR_{it}$), after subtracting the value of leisure for the employee from being out of work (L_{it}). Subtracting the net increase in costs (here zero) and discounting provides the net present value of return from preventing one nurse from being on sick leave for an entire calendar year (representing 222.5 working days). As neither employees or the hospital reported any net cost increases associated with implementing shift schedules with fewer short daily rest periods, this was excluded forn the calculations.

© 2025 Djupedal ILR et al. *JAMA Network Open.*

eTable 5. Elements in the Calculation of the Net Present Value (NPV) of Economic Returns for the Society From a Shift Schedule With a Reduced Number of Short Daily Rest Periods for Healthcare Workers Working Equivalent to ≥50% of a Full-Time Position [a]

	≥ 50% of a full-time position [b]		
% of full position	100%	50% [c]	75% [c]
Annual gross wage (Mean, nurses 2023)	630 000		
Payroll tax (14%)	88 200		
Mandatory pension plan (2%)	12 600		
Payroll tax (14%) on pension plan	1 764		
**Wi**: value of production	732 564	366 282	
Sickness benefits net of income tax (Gross wage*0,66)	415 800		
Income tax (34%) and payroll tax (14%)	304 164		
**TRi**: Reduction in transfers if working	719 964	359 982	
**λ**: Dead weight loss from taxation (20%)	143 993	71 996	
**Li**: Value of leisure when on sickness absence	120 582	60 291	
**r**: Discount rate	0,04		
Treatment effect (TE)[c]: $\left(\frac{\left(\Delta SA_{it}^{reduced\ SDRP} - \Delta SA_{it}^{usual\ SDRP}\right)}{5}\right) * 12$	-5,16	-6,82	-6,82
Extra costs from reduced SDRP shift schedule	0	0	0
NPV per person per year not on sickness absence:	726 899	363 449	545 174
NPV per person per day not on sickness absence (222,5 work days per year):	3 267	1 633	2 450
NPV from treatment of the treated (TE 5 months*value per day*# treated):	2 416 244	3 655 599	5 483 398
Annual NPV if treatment of all included in the project (TE 12 months*value per day*# healthcare workers):	13 671 448	19 640 029	29 460 044

© 2025 Djupedal ILR et al. *JAMA Network Open.*

Cost-benefit formula:

$$NPV = \sum_{i=1}^{n} \left\{ \sum_{t=1}^{Ti} \frac{\left(\Delta SA_{it}^{reduced\ SDRP} - \Delta SA_{it}^{usual\ SDRP}\right)(W_{it} - L_{it} + \lambda TR_{it}) - (1+\lambda)\left(C_{it}^{reduced\ SDRP} - C_{it}^{usual\ SDRP}\right)}{(1+r)^t} \right\}$$

NPV, Net Present Value; NOK, Norwegian Kroner; TE, Treatment Effect; ΔSA, difference in Sickness Absence; i, n treated; t, periods with treatment effect; SDRP, Short Daily Rest Period.

[a] The calculations follow the principles recommended by the Norwegian Ministry of Finance (R-109-2021).

[b] Assuming workers, over the calendar year, on average worked 50/75% of a full-time position.

[c] Based on treatment effects and numbers of treated, numbers of participants who worked ≥ 50% of full position.

[d] Treatment effects refer to the annual effect (in days) of implementing a shift schedule with a reduced number of short daily rest periods.

© 2025 Djupedal ILR et al. *JAMA Network Open.*

Possible unwanted negative effects or events

eTable 6. Total Number of Responses and Frequency Distribution to Questions About Possible Unwanted Negative Events or Effects as a Result of the Shift Schedule Over the Last Few Months. Responses Were Given at the End of the Six-Month Intervention Period (n=600). (Table continues on the next pages)

	**Shift schedule with minimal number of short daily rest periods** (intervention, n=316)	**Shift schedule maintaining the usual number of short daily rest periods** (control, n=284)
	n (%)	*n (%)*
I had more trouble sleeping		
Not at all	237 (75.0)	181 (63.7)
Little	32 (10.1)	40 (14.1)
Somewhat	23 (7.3)	36 (12.7)
To some extent	10 (3.2)	18 (6.3)
To a great extent	14 (4.4)	9 (3.2)
I became more stressed		
Not at all	230 (72.8)	205 (72.2)
Little	48 (15.2)	32 (11.3)
Somewhat	17 (5.4)	13 (4.6)
To some extent	14 (4.4)	13 (4.6)
To a great extent	7 (2.2)	4 (1.4)
I became more anxious/restless		
Not at all	275 (87.0)	231 (81.3)
Little	15 (4.7)	20 (7.0)
Somewhat	12 (3.8)	24 (8.5)
To some extent	11 (3.5)	7 (2.5)
To a great extent	3 (0.9)	2 (0.7)
I became more depressed/sad		
Not at all	274 (86.7)	225 (79.2)
Little	18 (5.7)	28 (9.9)
Somewhat	12 (3.8)	23 (8.1)
To some extent	9 (2.8)	6 (2.1)
To a great extent	3 (0.9)	2 (0.7)
I experienced greater hopelessness		
Not at all	263 (83.2)	236 (83.1)
Little	22 (7.0)	26 (9.2)
Somewhat	15 (4.7)	18 (6.3)
To some extent	13 (4.1)	2 (0.7)
To a great extent	3 (0.9)	2 (0.7)

[a] Questions were administered as a way for the participants to give feedback about the research project and to detect possible unwanted negative effects and events. The items do not represent outcomes in this trial and have therefore not been tested for significant differences between the groups.

© 2025 Djupedal ILR et al. *JAMA Network Open.*

eTable 6 continued (2/3). (Table continues on the next page)

	**Shift schedule with minimal number of short daily rest periods** (intervention, n=316)	**Shift schedule maintaining the usual number of short daily rest periods** (control, n=284)
	n (%)	*n (%)*
I experienced more unpleasant feelings		
Not at all	265 (83.9)	226 (79.6)
Little	27 (8.5)	29 (10.2)
Somewhat	8 (2.5)	24 (8.5)
To some extent	2 (3.8)	2 (0.7)
To a great extent	4 (1.3)	3 (1.1)
I experienced that my general condition worsened		
Not at all	276 (87.3)	228 (80.3)
Little	16 (5.1)	21 (7.4)
Somewhat	11 (3.5)	26 (9.2)
To some extent	11 (3.5)	5 (1.8)
To a great extent	2 (0.6)	4 (1.4)
I experienced less rest time between my work periods		
Not at all	251 (79.4)	203 (71.5)
Little	24 (7.6)	28 (9.9)
Somewhat	20 (6.3)	25 (8.8)
To some extent	11 (3.5)	18 (6.3)
To a great extent	10 (3.2)	10 (3.5)
I experienced a poorer balance between work and leisure		
Not at all	181 (57.3)	188 (66.2)
Little	52 (16.5)	37 (13.0)
Somewhat	37 (11.7)	31 (10.9)
To some extent	24 (7.6)	22 (7.7)
To a great extent	22 (7.0)	6 (2.1)
I experienced my shift schedule as more unfavourable		
Not at all	157 (49.7)	191 (67.3)
Little	63 (19.9)	40 (14.1)
Somewhat	28 (8.9)	33 (11.6)
To some extent	35 (11.1)	10 (3.5)
To a great extent	33 (10.4)	10 (3.5)
I experienced less flexibility in terms of being able to swap shifts		
Not at all	158 (50.0)	203 (71.5)
Little	55 (17.4)	41 (14.4)
Somewhat	32 (10.1)	23 (8.1)
To some extent	27 (8.5)	7 (2.5)
To a great extent	44 (13.9)	10 (3.5)
I had greater relational difficulties		
Not at all	269 (85.1)	241 (84.9)
Little	18 (5.7)	19 (6.7)
Somewhat	14 (4.4)	20 (7.0)
To some extent	11 (3.5)	3 (1.1)
To a great extent	4 (1.3)	1 (0.4)

© 2025 Djupedal ILR et al. *JAMA Network Open.*

eTable 6 continued (3/3)

	**Shift schedule with minimal number of short daily rest periods** (intervention, n=316)	**Shift schedule maintaining the usual number of short daily rest periods** (control, n=284)
	n (%)	*n (%)*
I experienced a worse psychosocial climate at work		
Not at all	234 (74.1)	229 (80.6)
Little	45 (14.2)	24 (8.5)
Somewhat	14 (4.4)	24 (8.5)
To some extent	13 (4.1)	5 (1.8)
To a great extent	10 (3.2)	2 (0.7)
I experienced that the quality of care for the patients deteriorated		
Not at all	231 (73.1)	228 (80.3)
Little	37 (11.7)	27 (9.5)
Somewhat	22 (7.0)	19 (6.7)
To some extent	17 (5.4)	8 (2.8)
To a great extent	9 (2.8)	2 (0.7)
I experienced that the continuity of care for the patients became worse		
Not at all	178 (56.3)	220 (77.5)
Little	55 (17.4)	27 (9.5)
Somewhat	35 (11.1)	22 (7.7)
To some extent	26 (8.2)	11 (3.9)
To a great extent	22 (7.0)	4 (1.4)
I found that I made more mistakes at work		
Not at all	289 (91.5)	247 (87.0)
Little	14 (4.4)	21 (7.4)
Somewhat	9 (2.8)	13 (4.6)
To some extent	4 (1.3)	2 (0.7)
To a great extent	0 (0.0)	1 (0.1)
I was less able to rest and recover when I had time off		
Not at all	225 (71.2)	210 (73.9)
Little	38 (12.0)	31 (10.9)
Somewhat	25 (7.9)	22 (7.7)
To some extent	16 (5.1)	15 (5.3)
To a great extent	12 (3.8)	6 (2.1)
I experienced greater difficulties in planning my family and leisure activities		
Not at all	193 (61.1)	205 (72.2)
Little	49 (15.5)	35 (12.3)
Somewhat	24 (7.6)	25 (8.8)
To some extent	27 (8.5)	13 (4.6)
To a great extent	23 (7.3)	6 (2.1)

© 2025 Djupedal ILR et al. *JAMA Network Open.*

eTable 7. Cluster-Adjusted Ordinal-Logistic Regression Results for Self-Reported Questions About Possible Unwanted Negative Events or Effects as a Result of the Shift Schedule the Last Few Months at Six-Month Follow-up (Intervention vs Control)

**Variables**	**OR**	**Estimate**	**SE**	**Wald *p*-value**	***p*-value Bonferroni**
I experienced my shift schedule as more unfavorable	2,13	0,75	0,18	**0,000**	**0,000**
I experienced less flexibility in terms of being able to swap shifts	2,58	0,95	0,25	**0,000**	**0,002**
I experienced that the continuity of care for the patients became worse	2,62	0,96	0,27	**0,000**	**0,006**
I had more trouble sleeping	0,57	-0,56	0,18	**0,002**	**0,032**
I became more depressed/sad	0,52	-0,65	0,26	0,013	0,229
I experienced greater difficulties in planning my family and leisure activities	1,62	0,48	0,20	0,015	0,270
I experienced less rest time between my work periods	0,60	-0,51	0,21	0,018	0,315
I experienced that my general condition worsened	0,55	-0,59	0,26	0,025	0,448
I became more anxious/restless	0,60	-0,52	0,26	0,048	0,858
I experienced a poorer balance between work and leisure	1,39	0,33	0,20	0,095	1,000
I experienced more unpleasant feelings	0,63	-0,46	0,29	0,113	1,000
I found that I made more mistakes at work	0,57	-0,56	0,36	0,115	1,000
I experienced that the quality of care for the patients deteriorated	1,41	0,35	0,27	0,199	1,000
I became more stressed	0,77	-0,26	0,24	0,281	1,000
I experienced a worse psychosocial climate at work	1,27	0,24	0,30	0,418	1,000
I experienced greater hopelessness	0,92	-0,09	0,25	0,721	1,000
I had greater relational difficulties	0,92	-0,08	0,26	0,765	1,000
I was less able to rest and recover when I had time off	1,03	0,03	0,21	0,883	1,000

Notes. Each odds ratio (OR) is derived from a cumulative-logit mixed model with hospital unit (clusters) as a random intercept and group (intervention = fewer short daily rest periods, control = usual schedule) as fixed effect. OR < 1 indicates lower odds of reporting a higher (more negative) response category in the intervention group; OR > 1 indicates higher odds. Nominal Wald p-values were adjusted for 18 comparisons using the Bonferroni method; only OR for which p<0.0028 remain statistically significant after correction (shown in bold). The p-value Bonferroni column shows the Wald p-values multiplied by 18 for reference.

© 2025 Djupedal ILR et al. *JAMA Network Open.*
