# Supplementary material for: Shift Schedule With Fewer Short Daily Rest Periods and Sickness Absence Among Health Care Workers: A Cluster Randomized Clinical Trial
Source: JAMA Netw Open. 2025 Sep 15;8(9):e2531568. doi: 10.1001/jamanetworkopen.2025.31568 (PMC12439055; doi:10.1001/jamanetworkopen.2025.31568)
Supplement: Supplement 3. — Data Sharing Statement [file jamanetwopen-e2531568-s003.pdf]

Data Sharing Statement

Djupedal. Effects of a Shift Schedule With Fewer Short Daily Rest Periods on Sickness Absence Among Health Care Workers. *JAMA Netw Open*. Published September 12, 2025. doi:10.1001/jamanetworkopen.2025.31568

Data
Additional Information: ClinicalTrials.gov number NCT04693182 (Registered December 31, 2020) https://clinicaltrials.gov/study/NCT04693182
Data available: No

Additional Information
Explanation for why data not available: The ethical approval of this trial does not allow for sharing of the individual-level data that support the findings. However, a dataset of synthetic data, generated based on the collected data, can be made available from the corresponding author upon reasonable request.
